# Supplementary material for: Myocardial infarction and stroke subsequent to urinary tract infection (MISSOURI): protocol for a self-controlled case series using linked electronic health records
Source: BMJ Open. 2022 Sep 22;12(9):e064586. doi: 10.1136/bmjopen-2022-064586 (PMC9511592; doi:10.1136/bmjopen-2022-064586)
Supplement: Supplementary data [file bmjopen-2022-064586supp001.pdf]

# Myocardial Infarction and stroke subsequent to urinary tract infection (MISSOURI): protocol for a self-controlled case series using linked electronic health records: Supplemental Material

## Supplemental Material A - ICD-10 Codes for Acute MI and Stroke to Identify Potential Cases

| Code | Description | Condition |
|---|---|---|
| I210 | Acute transmural myocardial infarction of anterior wall | MI |
| I211 | Acute transmural myocardial infarction of inferior wall | MI |
| I212 | Acute transmural myocardial infarction of other sites | MI |
| I213 | Acute transmural myocardial infarction of unspecified site | MI |
| I214 | Acute subendocardial myocardial infarction | MI |
| I219 | Acute myocardial infarction unspecified | MI |
| I220 | Subsequent myocardial infarction of anterior wall | MI |
| I221 | Subsequent myocardial infarction of inferior wall | MI |
| I228 | Subsequent myocardial infarction of other sites | MI |
| I229 | Subsequent myocardial infarction of unspecified site | MI |
| | | |
| I600 | Subarachnoid haemorrhage from carotid siphon and bifurcation | STROKE |
| I601 | Subarachnoid haemorrhage from middle cerebral artery | STROKE |
| I602 | Subarachnoid haemorrhage from anterior communicating artery | STROKE |
| I603 | Subarachnoid haemorrhage from posterior communicating artery | STROKE |
| I604 | Subarachnoid haemorrhage from basilar artery | STROKE |
| I605 | Subarachnoid haemorrhage from vertebral artery | STROKE |
| I606 | Subarachnoid haemorrhage from other intracranial arteries | STROKE |
| I607 | Subarachnoid haemorrhage from intracranial artery unspec | STROKE |
| I608 | Other subarachnoid haemorrhage | STROKE |
| I609 | Subarachnoid haemorrhage unspecified | STROKE |
| I610 | Intracerebral haemorrhage in hemisphere subcortical | STROKE |
| I611 | Intracerebral haemorrhage in hemisphere cortical | STROKE |
| I612 | Intracerebral haemorrhage in hemisphere unspecified | STROKE |
| I613 | Intracerebral haemorrhage in brain stem | STROKE |
| I614 | Intracerebral haemorrhage in cerebellum | STROKE |
| I615 | Intracerebral haemorrhage intraventricular | STROKE |
| I616 | Intracerebral haemorrhage multiple localized | STROKE |
| I618 | Other intracerebral haemorrhage | STROKE |
| I619 | Intracerebral haemorrhage unspecified | STROKE |
| I629 | Intracranial haemorrhage (nontraumatic)unspecified | STROKE |
| I630 | Cerebral infarct due to thrombosis of precerebral arteries | STROKE |
| I631 | Cerebral infarction due to embolism of precerebral arteries | STROKE |
| I632 | Cereb infarct due unsp occlusion or stenos precerebrl arts | STROKE |
| I633 | Cerebral infarction due to thrombosis of cerebral arteries | STROKE |
| I634 | Cerebral infarction due to embolism of cerebral arteries | STROKE |
| I635 | Cerebrl infarct due unspec occlusion or stenos cerebrl arts | STROKE |
| I636 | Cerebral infarction due to cerebral venous thrombosis, nonpyogenic | |
| I638 | Other cerebral infarction | STROKE |
| I639 | Cerebral infarction unspecified | STROKE |
| I64X | Stroke not specified as haemorrhage or infarction | STROKE |

## Supplemental Material B - Read Codes for UTI to Determine Risk Periods

| Code | Description |
|---|---|
| 1A1.. | Urinary frequency/Frequency of micturition/Micturition frequency/Polyuria |
| 1A12. | Frequency of micturition |
| 1A1Z. | Micturition frequency NOS |
| 1A44. | Urine looks cloudy |
| 1A45. | Blood in urine - haematuria/Blood in urine - symptom/Haematuria - symptom |
| 1A55. | Dysuria |
| 1AG.. | Recurrent urinary tract infections |
| 1AZ6. | Lower urinary tract symptoms |
| 1J4.. | Suspected UTI |
| K0A2. | Recurrent and persistent haematuria |
| K101. | Acute pyelonephritis |
| K101z | Acute pyelonephritis NOS |
| K10y0 | Pyelonephritis unspecified |
| K15.. | Cystitis |
| K150. | Acute cystitis |
| K152. | Other chronic cystitis |
| K152y | Chronic cystitis unspecified |
| K152z | Other chronic cystitis NOS |
| K155. | Recurrent cystitis |
| K15yz | Other cystitis NOS |
| K15z. | Cystitis NOS |
| K190. | Recurrent urinary tract infection/Urinary tract infection, site not specified |
| K1903 | Recurrent UTI/Recurrent urinary tract infection |
| K1905 | Urinary tract infection |
| K190z | Urinary tract infection, site not specified NOS |
| K1970 | Painless haematuria |
| K1971 | Painful haematuria |
| K1973 | Frank haematuria |
| Kyu51 | [X]Other cystitis |
| L1668 | Urinary tract infection complicating pregnancy |
| R08.. | [D]Urinary system symptoms |
| R081. | [D]Dysuria |
| R081z | [D]Dysuria NOS |
| R084. | [D]Micturition frequency and polyuria |
| R0840 | [D]Frequency of micturition, unspecified |
| R0842 | [D]Nocturia |
| R084z | [D]Frequency of micturition or polyuria NOS |
| R0908 | [D]Suprapubic pain |
| SP07Q | Catheter-associated urinary tract infection/CAUTI - catheter-associated urinary tract infection |

## Supplemental Material C - Read Codes for Antibiotics to Determine Risk Periods

| Code | Description |
|---|---|
| e31B. | *AMIX 250mg/5mL suspension |
| e319. | *AMOXIL SF 125mg/5mL syrup |
| e31a. | *AMOXIL SF 250mg/5mL syrup |
| e31d. | *AMOXIL SF 3g sachets |
| e31R. | *AMOXYMED 250mg capsules |
| e31i. | *AUGMENTIN 375mg disp tablets |
| e69e. | *CEPOREX 250mg/5mL suspension |
| eg68. | *CIPROXIN 100mg tablets |
| e31v. | *CO-AMOXICLAV 125mg/5mL susp |
| e31u. | *CO-AMOXICLAV 375mg disp tabs |
| egA3. | *FOSFOMYCIN 3g/sachet granules |
| eg17. | *MACRODANTIN 100mg capsules |
| eg16. | *MACRODANTIN 50mg capsules |
| eccb. | *MONOTRIM 50mg/5mL s/f susp |
| egA1. | *MONURIL 3g/sach granules |
| ecc3. | *TRIMETHOPRIM 300mg tablets |
| e3z5. | AMIX 250mg capsules |
| e3z6. | AMIX 500mg capsules |
| e3zo. | AMOXICILLIN 125mg/1.25mL susp |
| e3zk. | AMOXICILLIN 125mg/5mL s/f susp |
| e3zm. | AMOXICILLIN 125mg/5mL syrup |
| e311. | AMOXICILLIN 250mg capsules |
| e3zu. | AMOXICILLIN 250mg/5mL s/f susp |
| e3zn. | AMOXICILLIN 250mg/5mL syrup |
| e312. | AMOXICILLIN 500mg capsules |
| e3zq. | AMOXICILLIN powder 3g/sachet |
| e31b. | AMOXIL 125mg/1.25mL paed susp |
| e315. | AMOXIL 250mg capsules |
| e316. | AMOXIL 500mg capsules |
| e3zo. | AMOXYCILLIN 125mg/1.25mL susp |
| e3zk. | AMOXYCILLIN 125mg/5mL s/f susp |
| e3zm. | AMOXYCILLIN 125mg/5mL syrup |
| e311. | AMOXYCILLIN 250mg capsules |
| e3zu. | AMOXYCILLIN 250mg/5mL s/f susp |
| e3zn. | AMOXYCILLIN 250mg/5mL syrup |
| e312. | AMOXYCILLIN 500mg capsules |
| e3zq. | AMOXYCILLIN powder 3g/sachet |
| e31k. | AUGMENTIN 125/31 in 5mL susp |
| e31P. | AUGMENTIN 250/62 in 5mL susp |
| e31h. | AUGMENTIN 375mg tablets |
| e31T. | AUGMENTIN 625mg tablets |
| e31Y. | AUGMENTIN-DUO 400/57in5mL susp |
| e61C. | CEFACLOR 125mg/5mL s/f susp |
| e615. | CEFACLOR 125mg/5mL suspension |
| e614. | CEFACLOR 250mg capsules |
| e61D. | CEFACLOR 250mg/5mL s/f susp |
| e616. | CEFACLOR 250mg/5mL suspension |
| e61a. | CEFACLOR 375mg m/r tablets |
| e618. | CEFACLOR 500mg capsules |


| Code | Description |
|---|---|
| e31B. | *AMIX 250mg/5mL suspension |
| e319. | *AMOXIL SF 125mg/5mL syrup |
| e31a. | *AMOXIL SF 250mg/5mL syrup |
| e31d. | *AMOXIL SF 3g sachets |

| | |
|---|---|
| e31R. | *AMOXYMED 250mg capsules |
| e31i. | *AUGMENTIN 375mg disp tablets |
| e69e. | *CEPOREX 250mg/5mL suspension |
| eg68. | *CIPROXIN 100mg tablets |
| e31v. | *CO-AMOXICLAV 125mg/5mL susp |
| e31u. | *CO-AMOXICLAV 375mg disp tabs |
| egA3. | *FOSFOMYCIN 3g/sachet granules |
| eg17. | *MACRODANTIN 100mg capsules |
| eg16. | *MACRODANTIN 50mg capsules |
| eccb. | *MONOTRIM 50mg/5mL s/f susp |
| egA1. | *MONURIL 3g/sach granules |
| ecc3. | *TRIMETHOPRIM 300mg tablets |
| e3z5. | AMIX 250mg capsules |
| e3z6. | AMIX 500mg capsules |
| e3zo. | AMOXICILLIN 125mg/1.25mL susp |
| e3zk. | AMOXICILLIN 125mg/5mL s/f susp |
| e3zm. | AMOXICILLIN 125mg/5mL syrup |
| e311. | AMOXICILLIN 250mg capsules |
| e3zu. | AMOXICILLIN 250mg/5mL s/f susp |
| e3zn. | AMOXICILLIN 250mg/5mL syrup |
| e312. | AMOXICILLIN 500mg capsules |
| e3zq. | AMOXICILLIN powder 3g/sachet |
| e31b. | AMOXIL 125mg/1.25mL paed susp |
| e315. | AMOXIL 250mg capsules |
| e316. | AMOXIL 500mg capsules |
| e3zo. | AMOXYCILLIN 125mg/1.25mL susp |
| e3zk. | AMOXYCILLIN 125mg/5mL s/f susp |
| e3zm. | AMOXYCILLIN 125mg/5mL syrup |
| e311. | AMOXYCILLIN 250mg capsules |
| e3zu. | AMOXYCILLIN 250mg/5mL s/f susp |
| e3zn. | AMOXYCILLIN 250mg/5mL syrup |
| e312. | AMOXYCILLIN 500mg capsules |
| e3zq. | AMOXYCILLIN powder 3g/sachet |
| e31k. | AUGMENTIN 125/31 in 5mL susp |
| e31P. | AUGMENTIN 250/62 in 5mL susp |
| e31h. | AUGMENTIN 375mg tablets |
| e31T. | AUGMENTIN 625mg tablets |
| e31Y. | AUGMENTIN-DUO 400/57in5mL susp |
| e61C. | CEFACLOR 125mg/5mL s/f susp |
| e615. | CEFACLOR 125mg/5mL suspension |
| e614. | CEFACLOR 250mg capsules |
| e61D. | CEFACLOR 250mg/5mL s/f susp |
| e616. | CEFACLOR 250mg/5mL suspension |
| e61a. | CEFACLOR 375mg m/r tablets |
| e618. | CEFACLOR 500mg capsules |
| e69.. | CEFALEXIN |
| e695. | CEFALEXIN 125mg/5mL mixture |

| | |
|---|---|
| e69v. | CEFALEXIN 125mg/5mL syrup |
| e691. | CEFALEXIN 250mg capsules |
| e693. | CEFALEXIN 250mg tablets |
| e696. | CEFALEXIN 250mg/5mL mixture |
| e69w. | CEFALEXIN 250mg/5mL syrup |
| e692. | CEFALEXIN 500mg capsules |
| e694. | CEFALEXIN 500mg tablets |
| e697. | CEFALEXIN 500mg/5mL syrup |
| e69.. | CEPHALEXIN |
| e695. | CEPHALEXIN 125mg/5mL mixture |
| e69v. | CEPHALEXIN 125mg/5mL syrup |
| e691. | CEPHALEXIN 250mg capsules |
| e693. | CEPHALEXIN 250mg tablets |
| e696. | CEPHALEXIN 250mg/5mL mixture |
| e69w. | CEPHALEXIN 250mg/5mL syrup |
| e692. | CEPHALEXIN 500mg capsules |
| e694. | CEPHALEXIN 500mg tablets |
| e697. | CEPHALEXIN 500mg/5mL syrup |
| e69f. | CEPOREX 125mg/5mL syrup |
| e698. | CEPOREX 250mg capsules |
| e69a. | CEPOREX 250mg tablets |
| e69g. | CEPOREX 250mg/5mL syrup |
| e699. | CEPOREX 500mg capsules |
| e69b. | CEPOREX 500mg tablets |
| e69h. | CEPOREX 500mg/5mL syrup |
| eg6.. | CIPROFLOXACIN |
| eg67. | CIPROFLOXACIN 100mg tablets |
| eg6x. | CIPROFLOXACIN 250mg tablets |
| eg6w. | CIPROFLOXACIN 500mg tablets |
| eg69. | CIPROFLOXACIN 5g/100mL susp |
| eg6v. | CIPROFLOXACIN 750mg tablets |
| eg61. | CIPROXIN 250mg tablets |
| eg64. | CIPROXIN 500mg tablets |
| eg6A. | CIPROXIN 5g/100mL suspension |
| eg65. | CIPROXIN 750mg tablets |
| e31Q. | CO-AMOXICLAV 125/31mg/5mL susp |
| e31z. | CO-AMOXICLAV 250/62in5mL susp |
| e31t. | CO-AMOXICLAV 375mg tablets |
| e31X. | CO-AMOXICLAV 400/57mg susp |
| e31U. | CO-AMOXICLAV 625mg tablets |
| e612. | DISTACLOR 125mg/5mL suspension |
| e613. | DISTACLOR 250mg/5mL suspension |
| e617. | DISTACLOR 500mg capsules |
| e619. | DISTACLOR MR 375mg m/r tablets |
| ebI.. | FOSFOMYCIN |
| eg14. | FURADANTIN 100mg tablets |
| eg13. | FURADANTIN 50mg tablets |
| eg1C. | GENFURA 100mg tablets |

| | |
|---|---|
| eg1B. | GENFURA 50mg tablets |
| e69m. | KEFLEX 125mg/5mL suspension |
| e69i. | KEFLEX 250mg capsules |
| e69k. | KEFLEX 250mg tablets |
| e69n. | KEFLEX 250mg/5mL suspension |
| e69j. | KEFLEX 500mg capsules |
| e69l. | KEFLEX 500mg tablets |
| eg1A. | MACROBID 100mg m/r capsules |
| eg17. | MACRODANTIN 100mg capsules |
| eg16. | MACRODANTIN 50mg capsules |
| eg1x. | NITROFURANT 25mg/5mL s/f susp |
| eg1.. | NITROFURANTOIN |
| eg1z. | NITROFURANTOIN 100mg capsules |
| eg1w. | NITROFURANTOIN 100mg m/r caps |
| eg12. | NITROFURANTOIN 100mg tablets |
| eg1y. | NITROFURANTOIN 50mg capsules |
| eg11. | NITROFURANTOIN 50mg tablets |
| e52w. | PIVMECILLINAM HCL 200mg tabs |
| e521. | SELEXID 200mg tablets |
| ecc.. | TRIMETHOPRIM |
| ecc1. | TRIMETHOPRIM 100mg tablets |
| ecc2. | TRIMETHOPRIM 200mg tablets |
| ecc4. | TRIMETHOPRIM 50mg/5mL s/f susp |

**Supplemental Material D - ICD-10 Codes for UTI to Determine Risk Periods**

| Code | Description |
|---|---|
| N10 | Acute tubulo-interstitial nephritis |
| N12 | Tubulo-interstitial nephritis, not specified as acute or chronic |
| N30.0 | Acute cystitis |
| N30.8 | Other cystitis |
| N30.9 | Cystitis, unspecified |
| N39.0 | Urinary tract infection, site not specified |
