# Supplementary material for: Myocardial infarction and stroke subsequent to urinary tract infection (MISSOURI): protocol for a self-controlled case series using linked electronic health records
Source: BMJ Open. 2022 Sep 22;12(9):e064586. doi: 10.1136/bmjopen-2022-064586 (PMC9511592; doi:10.1136/bmjopen-2022-064586)
Supplement: Author's manuscript [file bmjopen-2022-064586.draft_revisions.pdf]

BMJ Open

BMJ Open is committed to open peer review. As part of this commitment we make the peer review history of every article we publish publicly available.

When an article is published we post the peer reviewers' comments and the authors' responses online. We also post the versions of the paper that were used during peer review. These are the versions that the peer review comments apply to.

The versions of the paper that follow are the versions that were submitted during the peer review process. They are not the versions of record or the final published versions. They should not be cited or distributed as the published version of this manuscript.

BMJ Open is an open access journal and the full, final, typeset and author-corrected version of record of the manuscript is available on our site with no access controls, subscription charges or pay-per-view fees (http://bmjopen.bmj.com).

If you have any questions on BMJ Open's open peer review process please email info.bmjopen@bmj.com

BMJ Open

Myocardial Infarction and stroke subsequent to urinary tract infection (MISSOURI): protocol for a self-controlled case series using linked electronic health records

Journal:	*BMJ Open*
Manuscript ID	bmjopen-2022-064586
Article Type:	Protocol
Date Submitted by the Author:	09-May-2022
Complete List of Authors:	Reeve, Nicola F; Cardiff University, Division of Population Medicine<br>Best, Victoria; Swansea University, Population Data Science<br>Gillespie, David; Cardiff University, Centre for Trials Research<br>Hughes, Kathryn; Cardiff University, PRIME Centre Wales, Division of Population Medicine<br>Lugg-Widger, Fiona; Cardiff University, Centre for Trials Research<br>Cannings-John, Rebecca; Cardiff University Centre for Trials Research,<br>Torabi, Fatemeh; Swansea University Medical School, Population Data Science<br>Wootton, Mandy; University Hospital of Wales Healthcare NHS Trust, Specialist Antimicrobial Chemotherapy Unit, Public Health Wales,<br>Akbari, Ashley; Swansea University Medical School,<br>Ahmed, Haroon; Cardiff University, Division of Population Medicine
Keywords:	Myocardial infarction < CARDIOLOGY, Stroke < NEUROLOGY, Urinary tract infections < UROLOGY

SCHOLARONE™
Manuscripts

Myocardial Infarction and stroke subsequent to urinary tract infection (MISSOURI): protocol for a self-controlled case series using linked electronic health records

Authors

Nicola F. Reeve[1], reeven1@cardiff.ac.uk

Victoria Best[2], v.e.e.best@swansea.ac.uk

David Gillespie[3], gillespied1@cardiff.ac.uk

Kathryn Hughes[4], hugheska6@cardiff.ac.uk

Fiona V. Lugg-Widger[3], luggfv@cardiff.ac.uk

Rebecca Cannings-John[3], canningsrl@Cardiff.ac.uk

Fatemeh Torabi[2], fatemeh.torabi@swansea.ac.uk

Mandy Wootton[5], mandy.wootton@wales.nhs.uk

Ashley Akbari[2], a.akbari@swansea.ac.uk

Haroon Ahmed[1], ahmedh2@cardiff.ac.uk

Corresponding author

Nicola F. Reeve

Division of Population Medicine

Cardiff University

3rd Floor, Neuadd Meirionnydd

Heath Park

Cardiff

CF14 4YS

Reeven1@cardiff.ac.uk

+44(0)29 20687895

Affiliations

[1] Division of Population Medicine, Cardiff University, Cardiff, UK

[2] Population Data Science, Swansea University Medical School, Swansea University, Swansea, UK

[3] Centre for Trials Research, Cardiff University, Cardiff, UK

For peer review only

[4] PRIME Centre Wales, Cardiff University School of Medicine, Division of Population Medicine, Cardiff, UK

[5] Specialist Antimicrobial Chemotherapy Unit, Public Health Wales, Microbiology Cardiff, University Hospital of Wales, Cardiff, UK

Keywords

Myocardial Infarction, Stroke, Urinary Tract Infection

Word Count

3,383

For peer review only

For peer review only - http://bmjopen.bmj.com/site/about/guidelines.xhtml

Abstract

Introduction There is increasing interest in the relationship between acute infections and acute cardiovascular events. Most previous research has focussed on understanding whether the risk of acute cardiovascular events increases following a respiratory tract infection (RTI). The relationship between Urinary Tract Infections (UTIs) and acute cardiovascular events is less well studied. Therefore, the aim of this study is to determine whether there is a causal relationship between UTI and acute Myocardial Infarction (MI) or stroke.

Methods and analysis We will undertake a self-controlled case series study using linked anonymised general practice, hospital admission, and microbiology data held within the Secure Anonymised Information Linkage (SAIL) Databank. Self-controlled case series is a relatively novel study design where individuals act as their own controls, thereby inherently controlling for time-invariant confounders. Only individuals who experience an exposure and outcome of interest are included.

We will identify individuals in the SAIL Databank who have a hospital admission record for acute MI or stroke during the study period of 2010-2020. Individuals will need to be aged 30-100 during the study period and be Welsh residents for inclusion. UTI will be identified using general practice, microbiology, and hospital admissions data. We will calculate the incidence of MI and stroke in pre-defined risk periods following a UTI and in “baseline” periods (without UTI exposure) and use conditional Poisson regression models to derive incidence rate ratios.

Ethics and dissemination Data access, research permissions and approvals have been obtained from the SAIL independent Information Governance Review Panel (IGRP), project number 0972. Findings will be disseminated through conferences, blogs, social media threads and peer-reviewed journals. Results will be of interest internationally to primary and secondary care clinicians who manage UTIs and may inform future clinical trials of preventative therapy.

Article Summary

Strengths and limitations of this study

- The self-controlled case series method controls for time-invariant confounding, enabling more reliable causal estimates of the association between UTI and acute MI or stroke, compared to between-individual study designs.
- A causal relationship between UTI and acute MI or stroke has implications for our understanding of cardiovascular disease mechanisms and may inform new methods of disease prevention.
- Using individual-level population-scale anonymised, routinely collected electronic health record (EHR) data provides adequate power to study sub-groups and maximises representativeness and generalisability.
- EHR data are collected and recorded for clinical purposes, and therefore the reliability of research findings is dependent on the quality and completeness of these data.
- Clinical and microbiological diagnoses of UTI are subject to caveats, and therefore we will use several definitions of UTI that utilise the different data sources in the SAIL Databank.

For peer review only - http://bmjopen.bmj.com/site/about/guidelines.xhtml

1 INTRODUCTION

Since the late 1990s, an increasing number of observational studies have found an association between acute infections and myocardial infarction (MI).[1-10] Most studies focused on respiratory tract infections (RTIs), and found an increased risk of acute MI in the 1-3 days following an RTI, with the effect size varying according to the infecting organism.[2–8] For example, Kwong et al. found a six-fold increase in the risk of MI in the week after influenza infection, a four-fold increase after respiratory syncytial virus and a three-fold increase after other respiratory viruses.[5] Several studies have also found evidence of an association between pneumonia and acute cardiovascular events (including MI and stroke).[9–13] The increased risk of acute cardiovascular events following pneumonia infection persists for up to 10 years.[9] This long-term risk of acute cardiovascular events has also been observed after other severe infections, including sepsis and bacteraemia.[14–17]

It is thought that acute infection may cause major cardiovascular events through three mechanisms. First, the inflammatory response from acute infection may destabilise atherosclerotic plaques. Second, the prothrombotic, procoagulant state associated with acute infection may increase the risk of thrombosis at the site of plaque disruption. Third, inflammation and fever lead to an increase in heart rate, which may cause "demand ischemia" if the metabolic demands of the myocardial cells exceed oxygen supply.[1]

Urinary tract infections (UTI) can affect any part of the urinary system, including the kidneys, ureters, bladder and urethra. Most infections involve the lower urinary tract: the bladder and the urethra. UTIs are common infections, with 37% of women reporting experiencing at least one in their lifetime, and 29% experiencing more than one.[18] UTIs are associated with considerable morbidity. The global burden of disease study 2010 estimates the disability-adjusted life years attributable to tubulointerstitial nephritis, pyelonephritis and UTI to be 45 (95% uncertainty interval 32-55) per 100,000 population.[19]

The relationship between UTIs and acute cardiovascular events is less well studied than for RTIs. Only one previous study has examined this relationship. Smeeth et al. used the self-controlled case series method to analyse data from the General Practice Research Database. They found increased rates of MI and stroke subsequent to UTI, with the risk being highest in the first three days.[6] However, the data analysed are almost 20 years old, and there have been no attempts to replicate the findings. Furthermore, the study defined UTI using clinical codes only, so it is unclear if the reported associations related to individuals with clinical symptoms alone, or symptoms and bacteriuria, making it difficult to interpret whether individuals had true UTIs, or whether non-specific symptoms were misdiagnosed as UTI but represented early signs of a cardiovascular event. In addition, other studies have found that roughly two-thirds of women suspected to have UTI on presentation to primary care have no evidence of UTI on microbiological culture.[20] Therefore, the use of clinical codes alone to define UTI may lead to bias from misclassification of the exposure.

Therefore, the aim of this study is to determine whether there is a causal relationship between UTI and acute MI or stroke by analysing linked general practice, hospital admission, and microbiology data, from a representative sample of the Welsh population. We will use the self-controlled case series method, which controls for time-invariant confounding, enabling us to more reliably draw

For peer review only - http://bmjopen.bmj.com/site/about/guidelines.xhtml

causal inferences between UTI and acute MI or stroke, compared to between-individual study designs.

2 METHODS AND ANALYSIS

2.1 Aims and Objectives

The specific objectives of this research are to:

1) Estimate incidence rate ratios for acute MI and stroke in the 90 days following a clinically suspected and microbiologically confirmed UTI compared to baseline (all times outside of the 90-day risk period).
2) Assess the effect of different methods of UTI ascertainment on estimated rate ratios (i.e. clinically suspected and microbiologically confirmed; clinically diagnosed only; clinically suspected but not supported by microbiology; and UTI diagnosed and treated in hospital).
3) Investigate whether the effect of a clinically suspected and microbiologically confirmed UTI on acute MI and stroke differs according to the infecting organism.

Our primary hypothesis is that a clinically suspected and microbiologically confirmed UTI will increase the risk of acute MI or stroke in the 0-90-day post-infection period.

2.2 Data

We will use the Secure Anonymised Information Linkage (SAIL) Databank. This is an internationally recognised trusted research environment (TRE), with robust secure storage, enabling access to anonymised, linkable, individual-level Welsh population-scale data for research, with a focus on improving population health and health services. Data within SAIL is pseudonymised and made available to approved projects and users following an application to, and approval from the independent information governance review panel (IGRP). SAIL's storage and linkage processes ensure anonymity: first, data sources being provided to SAIL are split as per the standard split file process, with the source organisation splitting the source data into demographic data and clinical data, with a system linkage field to allow data to be re-joined later. This addresses confidentiality and disclosure issues that arise when working with health data by separating easily recognised person-based variables such as name and date of birth from clinical data, including information on diagnoses, tests and prescriptions. The demographic data are anonymised and assigned an Anonymised Linkage Field (ALF). These split files are then joined together using the system linkage field by the SAIL team and made available to researchers following encryption.[21-23]

We will use the SAIL Databank to access the following linked data: Welsh Longitudinal General Practice data (WLGP), Patient Episode Database for Wales (PEDW), Welsh Results Reporting Service (WRRS). The WLGP contains data from 84% of general practices in Wales, consisting of longitudinal data for 2.6 million Welsh residents, representing 84% of the population.[24] Demographic data, clinical diagnoses and prescription data are included. The PEDW contains ICD-10 coded diagnoses for admissions to any Welsh hospital.[25] The WRRS contains data on all tests requested from primary and secondary care NHS Wales organisations processed and analysed in NHS Wales laboratories, including requests for urine microscopy, culture and antibiotic susceptibilities.[26] Data are available from the data sources at varying times based on when clinical information

For peer review only - http://bmjopen.bmj.com/site/about/guidelines.xhtml

systems began, with data quality improving over time. As such, for our study, based on our approvals and where the data sources have consistent coverage and quality, we will be using them between 1st January 2010 and 31st December 2020.

2.3 Study Design

Individuals who experience UTIs and individuals who do not can differ in unmeasured ways and hence could be sources of residual confounding. We will use the self-controlled case series (SCCS) design method to deal with this issue. The SCCS method is an epidemiological study design for which individuals act as their own control so that both measured and unmeasured characteristics that vary between individuals are completely controlled.[27] Only individuals who have experienced an outcome and exposure of interest are included. The SCCS method compares the incidence of an outcome during pre-defined risk periods with incidence during baseline periods (all times outside of risk and pre-risk periods) and estimates the temporal association between a transient exposure and outcome. Time-invariant covariates (e.g., sex) are inherently controlled for, and time varying covariates (e.g., age) are adjusted for within the models. The method was originally developed to investigate associations between vaccinations and acute adverse events, such as aseptic meningitis,[28, 29] but has since been applied in a range of epidemiological settings,[30–32] including non-acute events such as autism.[33] As with other study designs, the SCCS method makes several assumptions that need to be met in order to obtain valid and unbiased estimates, but there are model extensions which provide solutions to violations of these assumptions under certain circumstances.[34] The model assumptions, how they apply to our study, and the solutions to violations of the assumptions are given in Table 1.

A diagrammatic representation of observation time for an individual in the proposed SCCS design is given in Figure 1. Risk periods start on the date of a UTI and end 90 days later. The date of a UTI is the earliest date of occurrence of any of the events necessary for each different UTI definition. E.g., the definition of UTI in our primary analysis is a combination of a UTI-related diagnostic code and antibiotic prescription in WLGP data and a urine culture result that supports a diagnosis of UTI in the WRRS data, occurring within a 7-day window. In this case, the date of the UTI would be the date of whichever of the following events occurred first: UTI related diagnostic code, antibiotic prescription, supporting urine culture.

Individuals can have more than one UTI during the observation period, and therefore can have more than one 90-day risk period. Where risk periods overlap, the later period takes precedence, and the earlier period is shortened. There will be a pre-risk period of 7 days before the risk period, to allow for the situation where an individual has a UTI for several days prior to consultation, so events in the pre-risk period are not erroneously attributed to the baseline period.

Baseline periods are all times outside of the risk and pre-risk periods. The study period is from 1st January 2010 to 31st December 2020. The observation period is different for each individual. It generally follows the study period but may start later for some individuals who were not Welsh residents at the start of the study period but became so later, or who turned 30 years of age sometime during the study period. Similarly, the observation period may end before the study period when individuals moved out of Wales or died.

For peer review only - http://bmjopen.bmj.com/site/about/guidelines.xhtml

Table 1 Model assumptions and solutions to violations of those assumptions

Assumption	How the assumption applies to this study	Solution	Example of use of the solution in the literature
Subsequent exposures should not be affected by previous events.	We might see a temporary increase in UTIs subsequent to an MI or stroke event, which would bias estimates towards the null.	Apply a pre-risk period.	Gibson et al. studied the association between prescription drugs and road traffic accidents. As some drugs may be used to treat anxiety or pain caused by the crash, a 4-week pre-exposure period was included.[35]
	As both MI and stroke have relatively high death rates, the length of the observation period is dependent on events, and no further exposures are possible after death.	Use an event-dependent observation period model extension [36] and conduct a sensitivity analysis that repeats the analysis, excluding individuals who died within 30 days of the event.	Bruer et al. used the event-dependent observation period model extension in their study on the association between antipsychotic drugs and myocardial infarction.[37]<br>Langan et al. studied the risk of stroke following herpes zoster. They conducted a sensitivity analysis excluding individuals who died within 90 days of stroke.[38]
Event rates are constant within defined periods	MI and stroke are more common in older individuals and may be affected by seasonal changes.	Control for age and season effects.	Grave et al. studied the association between seasonal influenza vaccination and Guillan Barre syndrome. They adjusted for calendar month, as the vaccinations are seasonal by design.[39]<br>In a study of the association between chickenpox and stroke, Thomas et al. adjusted for age in 5-year age bands.[40]
Events are independently recurrent or rare.	MI and stroke are not independent: once an individual has a first event, they are more likely to have a second.	Study first events only.	Langan et al. began the observation period 12 months into follow-up time to ensure first stroke events had been correctly identified.[38]

For peer review only - http://bmjopen.bmj.com/site/about/guidelines.xhtml

2.4 Population

The SCCS method starts with identifying individuals who have had the outcome of interest. Therefore, the source population are individuals within the SAIL Databank who have a hospital admission record for acute MI or stroke during the study period. For inclusion, individuals will need to be aged 30-100 between 1st January 2010 and 31st December 2020 and be Welsh residents.

2.5 Outcomes

Outcomes of interest are acute MI or stroke, as identified by International Classification of Disease version 10 (ICD-10) codes from inpatient diagnoses recorded in the Patient Episode Database for Wales. A list of ICD-10 codes to be used are given in Supplemental Material A.

2.6 Exposure

The exposure of interest is UTI. The risk period is 0-90 days following a UTI. This period was chosen as previous research has shown an increase in acute MI and stroke risk in the 1-90 days following a UTI.[6] There will be a pre-risk period of seven days before the risk period, to allow for the situation where an individual has a UTI for several days prior to consultation, so events in this period are not erroneously attributed to the baseline period. Individuals can be exposed to a UTI more than once during the observation period. Each exposure will be followed by the same 90-day risk period. Baseline periods are all other times.

To ascertain UTI, we developed definitions that reflected the Public Health Wales Microbiology Division's standard operating procedure for the investigation of urine.[41] These procedures are followed by NHS microbiology laboratories across Wales. For each definition, the data sources required and the clinical scenario represented is summarised in Table 2, and the code lists used are given in Supplemental Material B-D. In our primary analysis, an individual will be regarded as being exposed to a UTI if the following events occur within a 7-day window:

1. A GP record of a UTI diagnostic or symptom code
2. A GP record of an antibiotic prescription
3. A microbiology record of a urine sample with bacterial growth of a single organism of >=10^8 colony forming units (cfu) per litre and white blood cells >=10^8 per litre. If there are two organisms grown, both must demonstrate growth of >=10^8 cfu per litre. More than two organisms will be regarded as mixed growth and thus not supportive of a UTI diagnosis. In a sensitivity analysis, we will widen the microbiological criteria and include all urine samples with bacterial growth of a single organism of >=10^7 colony forming units (cfu) per litre, irrespective of the white blood cell count.

In secondary analysis 1, we will estimate the risk of MI and stroke amongst individuals with a GP record of a UTI code and antibiotic prescription, and a microbiology record of a urine sample with mixed bacterial growth (any descriptor for 'mixed growth' or >3 organisms). This is an important analysis given the uncertain clinical significance of mixed bacterial growth in an individual with symptoms of UTI. In secondary analysis 2, an individual will be regarded as exposed to UTI with only a GP record of a diagnostic or symptom code and an antibiotic prescription (no microbiology). Secondary analysis 3 will estimate the risk of MI and stroke amongst individuals where UTI was suspected and treated by the GP, but urine microbiology showed bacterial growth of <10^7 cfu per litre (not supportive of a UTI diagnosis). Secondary analysis 4 will focus on individuals with a hospital admission with a UTI-related ICD-10 code and a microbiology record of a urine sample

For peer review only - http://bmjopen.bmj.com/site/about/guidelines.xhtml

with bacterial growth of a single organism of >=10^8 cfu per litre and white blood cells >=10^8 per litre. As for the primary analysis, if there are two organisms grown, both must demonstrate growth of >=10^8 cfu per litre, and more than two organisms will be regarded as mixed growth.

Table 2 Definitions of UTI for primary and secondary analyses. As the UTI definitions are combinations of two or more components, the start of the risk period is defined as the date of the earliest component

	**UTI-related Read code in GP data (Supplemental Material B)**	**Antibiotic prescription in GP data (Supplemental Material C)**	**UTI-related ICD-10 code in PEDW (Supplemental Material D)**	**Urine culture results in WRRS**	**Time frame**	**Clinical scenario**
**Primary analysis**	Yes	Yes	No	Yes, showing bacterial growth of >=10^8 cfu/L and WBC>= 10^8/L	Three codes occur within a 7-day window	GP clinically suspected and microbiologically confirmed UTI
**Secondary analysis 1**	Yes	Yes	No	Yes, showing mixed bacterial growth (any descriptor for 'mixed growth' or >3 organisms).	Three codes occur within a 7-day window	GP clinically suspected UTI with mixed growth
**Secondary analysis 2**	Yes	Yes	No	No	Same day	GP clinically diagnosed and treated UTI. It is important to consider this group as not all individuals with suspected UTI have urine culture, and limiting to those with culture is subject to selection bias.

For peer review only - http://bmjopen.bmj.com/site/about/guidelines.xhtml

**Secondary analysis 3**	Yes	Yes	No	Yes, showing bacterial growth of < 10^7 cfu per litre	Three codes occur within a 7-day window	UTI is clinically suspected but not supported by microbiology. This group is important to understand if early symptoms and signs of acute MI or stroke are attributed to UTI.
**Secondary analysis 4**	No	No	Yes	Yes, showing bacterial growth of >= 10^8 cfu/L and WBC>= 10^8/L	Two codes occur within a 7-day window	UTI diagnosed and/or treated in hospital

2.7 Statistical Analysis

We will describe the demographics of the study population, such as age and sex distribution, medical history, and prescribed medication, using means and standard deviations for continuous variables, and frequencies and proportions for categorical variables. For each analysis, we will use conditional logistic regression to estimate incidence rate ratios (IRRs), with 95% confidence intervals, for the risk of acute MI or stroke in pre-risk and risk periods compared to baseline periods. We will include only individuals who have experienced both the outcome and the exposure, and will include only the first acute MI or stroke diagnosis in the observation period. The IRRs will be adjusted for time-varying confounders: age, season and year of UTI diagnosis. Year of UTI diagnosis is included because diagnostic and coding practices may have changed over time as a result of guidance and awareness around microbial resistance. Adjusted IRRs will be reported for the risk of acute MI or stroke at 1-7, 8-14, 15-28 and 29-90 days after UTI. We will conduct the analysis in R, using the SCCS package. We will report the findings in accordance with RECORD and STROBE.

2.8 Sensitivity and sub-group analyses

We will perform several sensitivity and sub-group analyses to assess the robustness of the findings of our primary analysis to different assumptions:

- We will explore the impact of using a wider definition of MI and stroke, including ICD-10 codes for acute coronary syndromes and transient ischaemic attacks. This will include events that have potentially been missed by our main definition and assess how sensitive our findings are to the definition of MI and stroke.
- We will explore the impact of widening the microbiological definition of UTI to bacterial growth of a single organism of >= 10^7 cfu per litre irrespective of WBC count.
- We will differentiate first-ever MI or stroke from recurrent events, and report risk estimates separately. This analysis will exclude individuals with a PEDW record of an event before the observation period, and include only those who have their first ever event during the

For peer review only - http://bmjopen.bmj.com/site/about/guidelines.xhtml

observation period.

- We will extend the pre-risk period to 14 days. Some individuals may have had a UTI for several days prior to diagnosis, and so an acute MI or stroke during this time may relate to exposure but without a pre-risk period, would count towards the baseline period.
- We will repeat the analysis excluding individuals who died within 30 days of an event to examine the potential effect of an event dependent observation period.
- We will restrict the definition of UTI to include only antibiotic prescriptions for nitrofurantoin (currently recommended 1st line therapy) to explore whether the choice of antibiotic impacts the findings.
- We will examine whether the COVID-19 pandemic may affect our findings by (1) excluding individuals whose MI or stroke occurred in 2020, and (2) including an interaction term to explore whether the association between exposure and outcome differs in 2020 versus pre-2020.
- We will include an interaction term to explore whether the association between exposure and outcome differs in those with and without a history of diabetes, given its potential role as a risk factor of both UTI and MI/stroke.

2.9 Sample Size and Power

Our initial work has identified 51,656 individuals with acute MI and 58,146 with stroke in the SAIL Databank who meet all inclusion criteria. In the previous study by Smeeth et al.,[6] the sample size was 53,709 for acute MI, and 55,157 for stroke, where 16% of acute MI cases, and 21% of stroke cases were exposed to UTI.[6] Based on a conservative exposure rate of 10%, and an at-risk window of 90 days, we estimated the effect size that could be reliably detected with our potential sample size, using the sample size function in the SCCS package in R. The available sample provides 90% power to detect an incidence rate ratio (IRR) of 1.3 at the alpha = 0.05 level, which is smaller than the IRRs detected in Smeeth et al.[6]

2.10 Patient and Public Involvement

We developed this research proposal in collaboration with members of the Wales Centre for Primary and Emergency Care Research Service Users group (SUPER). We have a PPI plan and are consulting the SAIL consumer panel and SUPER regarding all stages of this research, including ongoing discussion of analysis plans, review of findings, and plans for dissemination (e.g. public facing outputs). We have extended our sub-group analysis to include individuals with diabetes in response to discussions with the SAIL consumer panel members. Identifying patient and public involvement for future stages of this research is a secondary objective of our PPI plan.

2.11 Summary of Cases

The individuals with an ICD-10 code for either MI or stroke were selected according to the eligibility criteria, as shown in Figure 2. There are 58,146 individuals with an ICD-10 code for stroke, and 51,656 individuals with an ICD-10 code for MI, a total of 105,930 unique individuals (it is possible for an individual to be in both the stroke and the MI group). Stroke cases were 49% male, MI cases 63% male. Female stroke cases were older than males, with a median age of 79 years (25th to 75th centiles 69-87) compared to a median of 74 years (25th to 75th centiles 64-82) for males. Female MI cases were also older, with a median age of 77 (25th to 75th centiles 66-85) compared to 69 (25th

For peer review only - http://bmjopen.bmj.com/site/about/guidelines.xhtml

to 75th centiles 59-78) for males.

The mean length of observation (study entry to study exit) of stroke cases was 2,796 days for females and 2,981 days for males. For MI cases, 2,981 for females and 3,251 for males. History of diagnoses and prescription drugs is taken from all health records available for each individual. Health data is available for a median of 13 years (25th to 75th centiles 8-16) prior to the study start.

Characteristics of cases, including a history of diagnoses and prescription drugs prior to an event, smoking status, Welsh Index of Multiple Deprivation (WIMD) version 2019 and electronic Frailty Index (eFI) are given in Table 3.

For peer review only

Table 3 Characteristics of cases of stroke and MI

Characteristic	Stroke	MI
Sex (% male)	49.4	62.8
Age of males (mean (25th, 75th centiles))	74 (64, 82)	69 (59, 78)
Age of females (mean (25th, 75th centiles))	79 (69, 87)	77 (66, 85)
Welsh Index of Multiple Deprivation (WIMD) 2019 quintiles*		
1 (least deprived)	19.8	20.6
2	20.8	20.5
3	20.3	20.9
4	20.4	20.1
5 (most deprived)	18.6	17.9
Current smoker (%)	19.8	24.2
Electronic frailty index (mean (sd))	0.16 (0.12)	0.15 (0.12)
Prescribed lipid lowering drugs (%)	49.0	49.6
Prescribed aspirin (%)	43.7	41.3
Prescribed hypertensive drugs (%)	59.1	55.1
Prescribed beta blockers (%)	39.6	37.3
Chronic Kidney Disease (%)	20.9	18.9
COPD (%)	9.9	10.9
Asthma (%)	15.1	16.0
Hypertension (%)	51.2	46.2
Diabetes (%)	19.4	20.7
Cardiovascular disease (%)	56.4	53.4
Coronary Heart Disease (%)	15.9	21.5
Atrial Fibrillation (%)	16.0	8.7
Heart Failure (%)	8.4	8.6
Peripheral Vascular Disease (%)	7.2	7.7
Angina (%)	11.3	16.0
Transient Ischaemic Attacks (%)	9.4	5.0
Total number of cases	58,150	51,659

* LSOA version 2011 and WIMD version 2019

For peer review only

2.12 Ethics and Dissemination

All study data will be held within the SAIL Databank, an ISO27001 certified trusted research environment (TRE) for anonymised individual-level data. Data access, research permissions and approvals have been obtained from the SAIL independent Information Governance Review Panel (IGRP), project number 0972. Analyses will be conducted within the SAIL TRE. There are strict disclosure control processes in place. Only aggregated outputs will be approved for release to ensure individuals are not identified. Findings will be disseminated through peer-review journals and conferences. Results will be of interest internationally to primary and secondary care clinicians who manage UTIs. An association between UTI and either MI or stroke will support a future funding application for a randomised trial of preventative treatments, such as antiplatelet drugs.

For peer review only - http://bmjopen.bmj.com/site/about/guidelines.xhtml

3 ACKNOWLEDGMENTS

This study makes use of anonymised data held in the Secure Anonymised Information Linkage (SAIL) Databank. We would like to acknowledge all the data providers who make anonymised data available for research.

4 AUTHOR CONTRIBUTIONS

HA is the chief investigator of the study. All authors have contributed to and are responsible for the final design of the study. NFR is responsible for study management. NFR, VB, FT and AA are responsible for the data management. NFR and RC-J are responsible for statistical planning and analysis. FLW is leading the patient and public involvement plans for the study. HA and MW are responsible for the microbiological definition of UTI. All authors have read and approved the final manuscript (NFR, VB, DG, KH, FLW, RC-J, FT, MW, AA, HA).

5 FUNDING STATEMENT

This work was supported by The British Heart Foundation, grant number PG/20/10419. The Centre for Trials Research is funded by Health and Care Research Wales and Cancer Research UK. Population Data Science, Swansea University is supported by Health Data Research UK (HDR-9006) and ADR Wales (grant ES/S007393/1). PRIME Centre Wales is funded by Health and Care Research Wales.

6 COMPETING INTERESTS STATEMENT

None declared

REFERENCES

[1] Musher DM, Abers MS, Corrales-Medina VF. Acute infection and myocardial infarction. *NEJM.* 2019;380(2):171-6.

[2] Warren-Gash C, Smeeth L, Hayward AC. Influenza as a trigger for acute myocardial infarction or death from cardiovascular disease: a systematic review. *Lancet Infect Dis*. 2009;9(10):601-10.

[3] Warren-Gash C, Hayward AC, Hemingway H, et al. Influenza infection and risk of acute myocardial infarction in England and 9 Wales: a CALIBER self-controlled case series study. *The Journal of infectious diseases.* 2012;206(11):1652-9.

[4] Warren-Gash C, Blackburn R, Whitaker H, et al. Laboratory-confirmed respiratory infections as triggers for acute myocardial infarction and stroke: a self-controlled case series analysis of national linked datasets from Scotland. *Eur Respir J*. 2018;51(3).

[5] Kwong JC, Schwartz KL, Campitelli MA, et al. Acute myocardial infarction after laboratory-confirmed influenza infection. *NEJM*. 2018;378(4):345-53.

For peer review only - http://bmjopen.bmj.com/site/about/guidelines.xhtml

[6] Smeeth L, Thomas SL, Hall AJ, et al. Risk of myocardial infarction and stroke after acute infection or vaccination. *NEJM*. 2004;351(25):2611-8.

[7] Clayton TC, Thompson M, Meade TW. Recent respiratory infection and risk of cardiovascular disease: case-control study through a general practice database. *Eur Heart J*. 2008;29(1):96-103.

[8] Warren-Gash C, Geretti AM, Hamilton G, et al. Influenza-like illness in acute myocardial infarction patients during the winter wave of the influenza A H1N1 pandemic in London: a case–control study. *BMJ Open*. 2013;3(5):e002604.

[9] Corrales-Medina VF, Alvarez KN, Weissfeld LA, et al. Association between hospitalization for pneumonia and subsequent risk of cardiovascular disease. *JAMA*. 2015;313(3):264-74.

[10] Corrales-Medina VF, Madjid M, Musher DM. Role of acute infection in triggering acute coronary syndromes. *Lancet Infect Dis*. 2010;10(2):83-92.

[11] Musher DM, Rueda AM, Kaka AS, et al. The association between pneumococcal pneumonia and acute cardiac events. *Clin Infect Dis*. 2007;45(2):158-65.

[12] Corrales-Medina VF, Serpa J, Rueda AM, et al. Acute bacterial pneumonia is associated with the occurrence of acute coronary syndromes. *Medicine.* 2009;88(3):154-9.

[13] Violi F, Cangemi R, Falcone M, et al. Cardiovascular complications and short-term mortality risk in community-acquired pneumonia. *Clin Infect Dis*. 2017;64(11):1486-93.

[14] Dalager-Pedersen M, Søgaard M, Schønheyder HC, et al. Risk for myocardial infarction and stroke after community-acquired bacteremia: a 20-year population-based cohort study. *Circulation*. 2014;129(13):1387-96. 10

[15] Bergh C, Fall K, Udumyan R, et al. Severe infections and subsequent delayed cardiovascular disease. *Eur J Prev Cardiol*. 2017;24(18):1958-66.

[16] Ou SM, Chu H, Chao PW, et al. Long-term mortality and major adverse cardiovascular events in sepsis survivors. A nationwide population-based study. *Am J Respir Crit Care Med.* 2016;194(2):209-17.

[17] Jafarzadeh SR, Thomas BS, Warren DK, et al. Longitudinal study of the effects of bacteremia and sepsis on 5-year risk of cardiovascular events. *Rev Infect Dis*. 2016;63(4):495-500.

[18] Butler CC, Hawking MK, Quigley A, et al. Incidence, severity, help seeking, and management of uncomplicated urinary tract infection: a population-based survey. *Br J Gen Pract*. 2015;65(639):e702-7.

[19] Murray CJ, Vos T, Lozano R, et al. Disability-adjusted life years (DALYs) for 291 diseases and injuries in 21 regions, 1990–2010: a systematic analysis for the Global Burden of Disease Study 2010. *Lancet*. 2012;380(9859):2197-223.

For peer review only - http://bmjopen.bmj.com/site/about/guidelines.xhtml

[20] Butler CC, Francis N, Thomas-Jones E, et al. Variations in presentation, management, and patient outcomes of urinary tract infection: a prospective four-country primary care observational cohort study. *Br J Gen Pract*. 2017;67(665):e830-41.

[21] Jones KH, Ford DV, Thompson S, et al. A profile of the SAIL databank on the UK secure research platform. *Int J Popul Data Sci.* 2019;4(2).

[22] Lyons RA, Jones KH, John G, et al. The SAIL databank: linking multiple health and social care datasets. *BMC Med Inform Decis Mak*. 2009;9(1):1-8.

[23] Ford DV, Jones KH, Verplancke JP, et al. The SAIL Databank: building a national architecture for e-health research and evaluation. *BMC Health Serv Res.* 2009;9(1):1-12.

[24] SAIL. Welsh Longitudinal GP Dataset - Welsh Primary Care (WLGP); 2021. Available from: https://web.www.healthdatagateway.org/dataset/ 33fc3ffd-aa4c-4a16-a32f-0c900aaea3d2.

[25] SAIL. Patient Episode Dataset for Wales (PEDW); 2021. Available from: https://web.www.healthdatagateway.org/dataset/ 4c33a5d2-164c-41d7-9797-dc2b008cc852.

[26] SAIL. Welsh Results Reports Service (WRRS); 2021. Available from: https://web. www.healthdatagateway.org/dataset/71d37610-ac55-432d-82a3-bdb04407acd8.

[27] Farrington P, Whitaker H, Weldeselassie YG. Self-controlled case series studies: a modelling guide with R. Chapman and Hall/CRC; 2018.

[28] Miller E, Farrington P, Goldracre M, et al. Risk of aseptic meningitis after measles, mumps, and rubella vaccine in UK children. *Lancet.* 1993;341(8851):979-82.

[29] Farrington P, Rush M, Miller E, et al. A new method for active surveillance of adverse events from diphtheria/tetanus/pertussis and measles/mumps/rubella vaccines. *Lancet.* 1995;345(8949):567-9.

[30] Whitaker HJ, Paddy Farrington C, Spiessens B, et al. Tutorial in biostatistics: the self-controlled case series method. *Stat Med.* 2006;25(10):1768-97.

[31] Douglas IJ, Smeeth L. Exposure to antipsychotics and risk of stroke: self controlled case series study. *BMJ*. 2008;337.

[32] Douglas IJ, Evans SJ, Pocock S, et al. The risk of fractures associated with thiazolidinediones: a self-controlled case-series study. *PLoS Med*. 2009;6(9):e1000154.

[33] Taylor B, Miller E, Farrington C, et al. Autism and measles, mumps, and rubella vaccine: no epidemiological evidence for a causal association. *Lancet*. 1999;353(9169):2026-9.

[34] Petersen I, Douglas I, Whitaker H. Self controlled case series methods: an alternative to standard epidemiological study designs. *BMJ.* 2016;354.

[35] Gibson JE, Hubbard RB, Smith CJ, et al. Use of self-controlled analytical techniques to assess

For peer review only - http://bmjopen.bmj.com/site/about/guidelines.xhtml

the association between use of prescription medications and the risk of motor vehicle crashes. *Am J Epidemiol.* 2009;169(6):761-8.

[36] Farrington CP, Anaya-Izquierdo K, Whitaker HJ, et al. Self-controlled case series analysis with event-dependent observation periods. *J Am Stat Assoc*. 2011;106(494):417-26.

[37] Brauer R, Smeeth L, Anaya-Izquierdo K, et al. Antipsychotic drugs and risks of myocardial infarction: a self-controlled case series study. *Eur Heart J*. 2015;36(16):984-92.

[38] Langan SM, Minassian C, Smeeth L, et al. Risk of stroke following herpes zoster: a self-controlled case-series study. *Clin Infect Dis*. 2014;58(11):1497-503.

[39] Grave C, Boucheron P, Rudant J, et al. Seasonal influenza vaccine and Guillain-Barré syndrome: a self-controlled case series study. *Neurology*. 2020;94(20):e2168-79. 12

[40] Thomas SL, Minassian C, Ganesan V, et al. Chickenpox and risk of stroke: a self-controlled case series analysis. *Clin Infect Dis*. 2014;58(1):61-8.

[41] Microbiology Division Public Health Wales. CDBBA 041 Bench Aid: Investigation of Urine; 2021.

For peer review only

For peer review only - http://bmjopen.bmj.com/site/about/guidelines.xhtml

FIGURE LEGENDS

Figure 1 Diagrammatic representation of observation time for an individual in the proposed Self-Controlled Case Series design

Figure 2: Case selection process. *Individuals appear and are counted in both the stroke and MI datasets if they had both an MI and a stroke event within the study period.

For peer review only

For peer review only - http://bmjopen.bmj.com/site/about/guidelines.xhtml

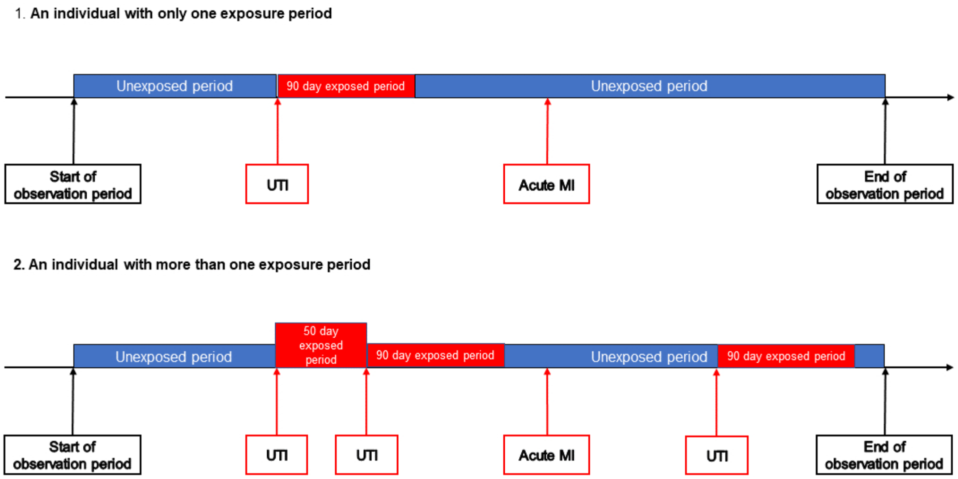

Diagrammatic representation of observation time for an individual in the proposed Self-Controlled Case Series design

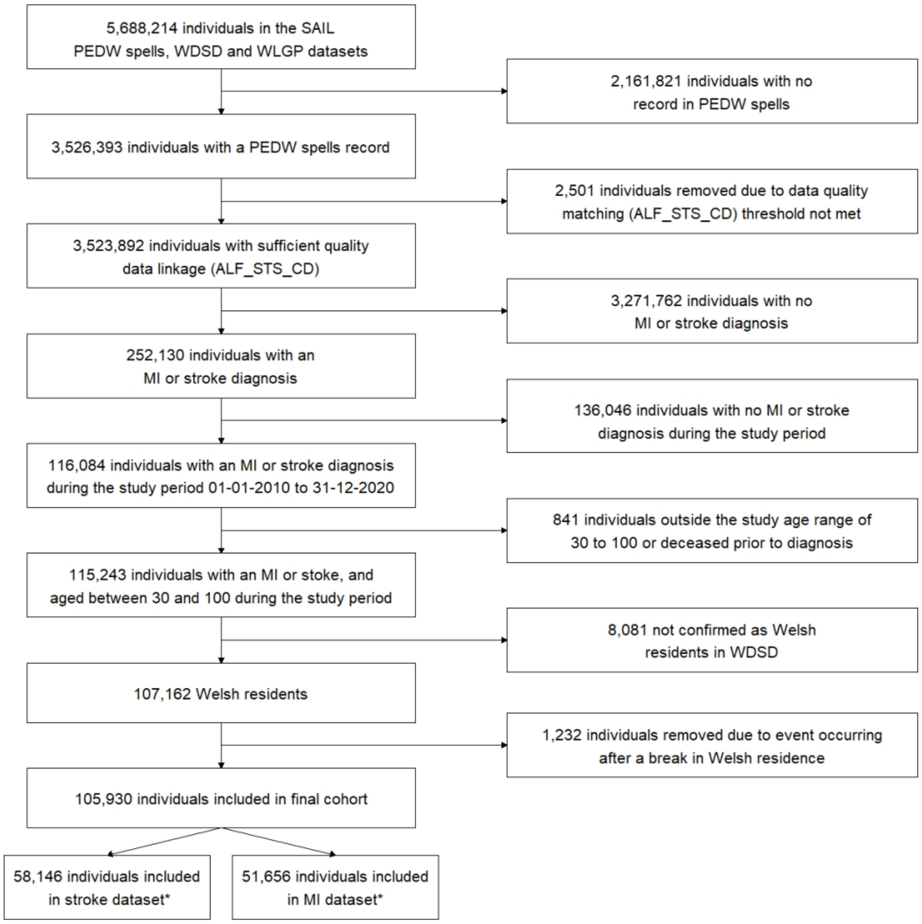

Case selection process. *Individuals appear and are counted in both the stroke and MI datasets if they had both an MI and a stroke event within the study period.

Myocardial Infarction and stroke subsequent to urinary tract infection (MISSOURI): protocol for a self-controlled case series using linked electronic health records: Supplemental Material

Supplemental Material A - ICD-10 Codes for Acute MI and Stroke to Identify Potential Cases

Code	Description	Condition
I210	Acute transmural myocardial infarction of anterior wall	MI
I211	Acute transmural myocardial infarction of inferior wall	MI
I212	Acute transmural myocardial infarction of other sites	MI
I213	Acute transmural myocardial infarction of unspecified site	MI
I214	Acute subendocardial myocardial infarction	MI
I219	Acute myocardial infarction unspecified	MI
I220	Subsequent myocardial infarction of anterior wall	MI
I221	Subsequent myocardial infarction of inferior wall	MI
I228	Subsequent myocardial infarction of other sites	MI
I229	Subsequent myocardial infarction of unspecified site	MI
I600	Subarachnoid haemorrhage from carotid siphon and bifurcation	STROKE
I601	Subarachnoid haemorrhage from middle cerebral artery	STROKE
I602	Subarachnoid haemorrhage from anterior communicating artery	STROKE
I603	Subarachnoid haemorrhage from posterior communicating artery	STROKE
I604	Subarachnoid haemorrhage from basilar artery	STROKE
I605	Subarachnoid haemorrhage from vertebral artery	STROKE
I606	Subarachnoid haemorrhage from other intracranial arteries	STROKE
I607	Subarachnoid haemorrhage from intracranial artery unspec	STROKE
I608	Other subarachnoid haemorrhage	STROKE
I609	Subarachnoid haemorrhage unspecified	STROKE
I610	Intracerebral haemorrhage in hemisphere subcortical	STROKE
I611	Intracerebral haemorrhage in hemisphere cortical	STROKE
I612	Intracerebral haemorrhage in hemisphere unspecified	STROKE
I613	Intracerebral haemorrhage in brain stem	STROKE
I614	Intracerebral haemorrhage in cerebellum	STROKE
I615	Intracerebral haemorrhage intraventricular	STROKE
I616	Intracerebral haemorrhage multiple localized	STROKE
I618	Other intracerebral haemorrhage	STROKE
I619	Intracerebral haemorrhage unspecified	STROKE
I629	Intracranial haemorrhage (nontraumatic)unspecified	STROKE
I630	Cerebral infarct due to thrombosis of precerebral arteries	STROKE
I631	Cerebral infarction due to embolism of precerebral arteries	STROKE
I632	Cereb infarct due unsp occlusion or stenos precerebrl arts	STROKE
I633	Cerebral infarction due to thrombosis of cerebral arteries	STROKE
I634	Cerebral infarction due to embolism of cerebral arteries	STROKE
I635	Cerebrl infarct due unspec occlusion or stenos cerebrl arts	STROKE
I636	Cerebral infarction due to cerebral venous thrombosis, nonpyogenic	
I638	Other cerebral infarction	STROKE
I639	Cerebral infarction unspecified	STROKE
I64X	Stroke not specified as haemorrhage or infarction	STROKE

For peer review only

Supplemental Material B - Read Codes for UTI to Determine Risk Periods

Code	Description
1A1..	Urinary frequency/Frequency of micturition/Micturition frequency/Polyuria
1A12.	Frequency of micturition
1A1Z.	Micturition frequency NOS
1A44.	Urine looks cloudy
1A45.	Blood in urine - haematuria/Blood in urine - symptom/Haematuria - symptom
1A55.	Dysuria
1AG..	Recurrent urinary tract infections
1AZ6.	Lower urinary tract symptoms
1J4..	Suspected UTI
K0A2.	Recurrent and persistent haematuria
K101.	Acute pyelonephritis
K101z	Acute pyelonephritis NOS
K10y0	Pyelonephritis unspecified
K15..	Cystitis
K150.	Acute cystitis
K152.	Other chronic cystitis
K152y	Chronic cystitis unspecified
K152z	Other chronic cystitis NOS
K155.	Recurrent cystitis
K15yz	Other cystitis NOS
K15z.	Cystitis NOS
K190.	Recurrent urinary tract infection/Urinary tract infection, site not specified
K1903	Recurrent UTI/Recurrent urinary tract infection
K1905	Urinary tract infection
K190z	Urinary tract infection, site not specified NOS
K1970	Painless haematuria
K1971	Painful haematuria
K1973	Frank haematuria
Kyu51	[X]Other cystitis
L1668	Urinary tract infection complicating pregnancy
R08..	[D]Urinary system symptoms
R081.	[D]Dysuria
R081z	[D]Dysuria NOS
R084.	[D]Micturition frequency and polyuria
R0840	[D]Frequency of micturition, unspecified
R0842	[D]Nocturia
R084z	[D]Frequency of micturition or polyuria NOS
R0908	[D]Suprapubic pain
SP07Q	Catheter-associated urinary tract infection/CAUTI - catheter-associated urinary tract infection

Supplemental Material C - Read Codes for Antibiotics to Determine Risk Periods

For peer review only

Code	Description
e31B.	*AMIX 250mg/5mL suspension
e319.	*AMOXIL SF 125mg/5mL syrup
e31a.	*AMOXIL SF 250mg/5mL syrup
e31d.	*AMOXIL SF 3g sachets
e31R.	*AMOXYMED 250mg capsules
e31i.	*AUGMENTIN 375mg disp tablets
e69e.	*CEPOREX 250mg/5mL suspension
eg68.	*CIPROXIN 100mg tablets
e31v.	*CO-AMOXICLAV 125mg/5mL susp
e31u.	*CO-AMOXICLAV 375mg disp tabs
egA3.	*FOSFOMYCIN 3g/sachet granules
eg17.	*MACRODANTIN 100mg capsules
eg16.	*MACRODANTIN 50mg capsules
eccb.	*MONOTRIM 50mg/5mL s/f susp
egA1.	*MONURIL 3g/sach granules
ecc3.	*TRIMETHOPRIM 300mg tablets
e3z5.	AMIX 250mg capsules
e3z6.	AMIX 500mg capsules
e3zo.	AMOXICILLIN 125mg/1.25mL susp
e3zk.	AMOXICILLIN 125mg/5mL s/f susp
e3zm.	AMOXICILLIN 125mg/5mL syrup
e311.	AMOXICILLIN 250mg capsules
e3zu.	AMOXICILLIN 250mg/5mL s/f susp
e3zn.	AMOXICILLIN 250mg/5mL syrup
e312.	AMOXICILLIN 500mg capsules
e3zq.	AMOXICILLIN powder 3g/sachet
e31b.	AMOXIL 125mg/1.25mL paed susp
e315.	AMOXIL 250mg capsules
e316.	AMOXIL 500mg capsules
e3zo.	AMOXYCILLIN 125mg/1.25mL susp
e3zk.	AMOXYCILLIN 125mg/5mL s/f susp
e3zm.	AMOXYCILLIN 125mg/5mL syrup
e311.	AMOXYCILLIN 250mg capsules
e3zu.	AMOXYCILLIN 250mg/5mL s/f susp
e3zn.	AMOXYCILLIN 250mg/5mL syrup
e312.	AMOXYCILLIN 500mg capsules
e3zq.	AMOXYCILLIN powder 3g/sachet
e31k.	AUGMENTIN 125/31 in 5mL susp
e31P.	AUGMENTIN 250/62 in 5mL susp
e31h.	AUGMENTIN 375mg tablets
e31T.	AUGMENTIN 625mg tablets
e31Y.	AUGMENTIN-DUO 400/57in5mL susp
e61C.	CEFACLOR 125mg/5mL s/f susp
e615.	CEFACLOR 125mg/5mL suspension
e614.	CEFACLOR 250mg capsules
e61D.	CEFACLOR 250mg/5mL s/f susp
e616.	CEFACLOR 250mg/5mL suspension
e61a.	CEFACLOR 375mg m/r tablets
e618.	CEFACLOR 500mg capsules

Code	Description
e31B.	*AMIX 250mg/5mL suspension
e319.	*AMOXIL SF 125mg/5mL syrup
e31a.	*AMOXIL SF 250mg/5mL syrup
e31d.	*AMOXIL SF 3g sachets

For peer review only

e31R.	*AMOXYMED 250mg capsules
e31i.	*AUGMENTIN 375mg disp tablets
e69e.	*CEPOREX 250mg/5mL suspension
eg68.	*CIPROXIN 100mg tablets
e31v.	*CO-AMOXICLAV 125mg/5mL susp
e31u.	*CO-AMOXICLAV 375mg disp tabs
egA3.	*FOSFOMYCIN 3g/sachet granules
eg17.	*MACRODANTIN 100mg capsules
eg16.	*MACRODANTIN 50mg capsules
eccb.	*MONOTRIM 50mg/5mL s/f susp
egA1.	*MONURIL 3g/sach granules
ecc3.	*TRIMETHOPRIM 300mg tablets
e3z5.	AMIX 250mg capsules
e3z6.	AMIX 500mg capsules
e3zo.	AMOXICILLIN 125mg/1.25mL susp
e3zk.	AMOXICILLIN 125mg/5mL s/f susp
e3zm.	AMOXICILLIN 125mg/5mL syrup
e311.	AMOXICILLIN 250mg capsules
e3zu.	AMOXICILLIN 250mg/5mL s/f susp
e3zn.	AMOXICILLIN 250mg/5mL syrup
e312.	AMOXICILLIN 500mg capsules
e3zq.	AMOXICILLIN powder 3g/sachet
e31b.	AMOXIL 125mg/1.25mL paed susp
e315.	AMOXIL 250mg capsules
e316.	AMOXIL 500mg capsules
e3zo.	AMOXYCILLIN 125mg/1.25mL susp
e3zk.	AMOXYCILLIN 125mg/5mL s/f susp
e3zm.	AMOXYCILLIN 125mg/5mL syrup
e311.	AMOXYCILLIN 250mg capsules
e3zu.	AMOXYCILLIN 250mg/5mL s/f susp
e3zn.	AMOXYCILLIN 250mg/5mL syrup
e312.	AMOXYCILLIN 500mg capsules
e3zq.	AMOXYCILLIN powder 3g/sachet
e31k.	AUGMENTIN 125/31 in 5mL susp
e31P.	AUGMENTIN 250/62 in 5mL susp
e31h.	AUGMENTIN 375mg tablets
e31T.	AUGMENTIN 625mg tablets
e31Y.	AUGMENTIN-DUO 400/57in5mL susp
e61C.	CEFACLOR 125mg/5mL s/f susp
e615.	CEFACLOR 125mg/5mL suspension
e614.	CEFACLOR 250mg capsules
e61D.	CEFACLOR 250mg/5mL s/f susp
e616.	CEFACLOR 250mg/5mL suspension
e61a.	CEFACLOR 375mg m/r tablets
e618.	CEFACLOR 500mg capsules
e69..	CEFALEXIN
e695.	CEFALEXIN 125mg/5mL mixture

For peer review only

e69v.	CEFALEXIN 125mg/5mL syrup
e691.	CEFALEXIN 250mg capsules
e693.	CEFALEXIN 250mg tablets
e696.	CEFALEXIN 250mg/5mL mixture
e69w.	CEFALEXIN 250mg/5mL syrup
e692.	CEFALEXIN 500mg capsules
e694.	CEFALEXIN 500mg tablets
e697.	CEFALEXIN 500mg/5mL syrup
e69..	CEPHALEXIN
e695.	CEPHALEXIN 125mg/5mL mixture
e69v.	CEPHALEXIN 125mg/5mL syrup
e691.	CEPHALEXIN 250mg capsules
e693.	CEPHALEXIN 250mg tablets
e696.	CEPHALEXIN 250mg/5mL mixture
e69w.	CEPHALEXIN 250mg/5mL syrup
e692.	CEPHALEXIN 500mg capsules
e694.	CEPHALEXIN 500mg tablets
e697.	CEPHALEXIN 500mg/5mL syrup
e69f.	CEPOREX 125mg/5mL syrup
e698.	CEPOREX 250mg capsules
e69a.	CEPOREX 250mg tablets
e69g.	CEPOREX 250mg/5mL syrup
e699.	CEPOREX 500mg capsules
e69b.	CEPOREX 500mg tablets
e69h.	CEPOREX 500mg/5mL syrup
eg6..	CIPROFLOXACIN
eg67.	CIPROFLOXACIN 100mg tablets
eg6x.	CIPROFLOXACIN 250mg tablets
eg6w.	CIPROFLOXACIN 500mg tablets
eg69.	CIPROFLOXACIN 5g/100mL susp
eg6v.	CIPROFLOXACIN 750mg tablets
eg61.	CIPROXIN 250mg tablets
eg64.	CIPROXIN 500mg tablets
eg6A.	CIPROXIN 5g/100mL suspension
eg65.	CIPROXIN 750mg tablets
e31Q.	CO-AMOXICLAV 125/31mg/5mL susp
e31z.	CO-AMOXICLAV 250/62in5mL susp
e31t.	CO-AMOXICLAV 375mg tablets
e31X.	CO-AMOXICLAV 400/57mg susp
e31U.	CO-AMOXICLAV 625mg tablets
e612.	DISTACLOR 125mg/5mL suspension
e613.	DISTACLOR 250mg/5mL suspension
e617.	DISTACLOR 500mg capsules
e619.	DISTACLOR MR 375mg m/r tablets
ebl..	FOSFOMYCIN
eg14.	FURADANTIN 100mg tablets
eg13.	FURADANTIN 50mg tablets
eg1C.	GENFURA 100mg tablets

For peer review only

eg1B.	GENFURA 50mg tablets
e69m.	KEFLEX 125mg/5mL suspension
e69i.	KEFLEX 250mg capsules
e69k.	KEFLEX 250mg tablets
e69n.	KEFLEX 250mg/5mL suspension
e69j.	KEFLEX 500mg capsules
e69l.	KEFLEX 500mg tablets
eg1A.	MACROBID 100mg m/r capsules
eg17.	MACRODANTIN 100mg capsules
eg16.	MACRODANTIN 50mg capsules
eg1x.	NITROFURANT 25mg/5mL s/f susp
eg1..	NITROFURANTOIN
eg1z.	NITROFURANTOIN 100mg capsules
eg1w.	NITROFURANTOIN 100mg m/r caps
eg12.	NITROFURANTOIN 100mg tablets
eg1y.	NITROFURANTOIN 50mg capsules
eg11.	NITROFURANTOIN 50mg tablets
e52w.	PIVMECILLINAM HCL 200mg tabs
e521.	SELEXID 200mg tablets
ecc..	TRIMETHOPRIM
ecc1.	TRIMETHOPRIM 100mg tablets
ecc2.	TRIMETHOPRIM 200mg tablets
ecc4.	TRIMETHOPRIM 50mg/5mL s/f susp

For peer review only

Supplemental Material D - ICD-10 Codes for UTI to Determine Risk Periods

Code	Description
N10	Acute tubulo-interstitial nephritis
N12	Tubulo-interstitial nephritis, not specified as acute or chronic
N30.0	Acute cystitis
N30.8	Other cystitis
N30.9	Cystitis, unspecified
N39.0	Urinary tract infection, site not specified

For peer review only

Myocardial Infarction and stroke subsequent to urinary tract infection (MISSOURI): protocol for a self-controlled case series using linked electronic health records

Journal:	*BMJ Open*
Manuscript ID	bmjopen-2022-064586.R1
Article Type:	Protocol
Date Submitted by the Author:	17-Aug-2022

Complete List of Authors:	Reeve, Nicola F; Cardiff University, Division of Population Medicine<br>Best, Victoria; Swansea University, Population Data Science<br>Gillespie, David; Cardiff University, Centre for Trials Research<br>Hughes, Kathryn; Cardiff University, PRIME Centre Wales, Division of Population Medicine<br>Lugg-Widger, Fiona; Cardiff University, Centre for Trials Research<br>Cannings-John, Rebecca; Cardiff University Centre for Trials Research,<br>Torabi, Fatemeh; Swansea University Medical School, Population Data Science<br>Wootton, Mandy; University Hospital of Wales Healthcare NHS Trust, Specialist Antimicrobial Chemotherapy Unit, Public Health Wales,<br>Akbari, Ashley; Swansea University Medical School,<br>Ahmed, Haroon; Cardiff University, Division of Population Medicine

**Primary Subject Heading**:	Infectious diseases
Secondary Subject Heading:	Cardiovascular medicine
Keywords:	Myocardial infarction < CARDIOLOGY, Stroke < NEUROLOGY, Urinary tract infections < UROLOGY

Myocardial Infarction and stroke subsequent to urinary tract infection (MISSOURI): protocol for a self-controlled case series using linked electronic health records

Authors

Nicola F. Reeve[1], reeven1@cardiff.ac.uk

Victoria Best[2], v.e.e.best@swansea.ac.uk

David Gillespie[3], gillespied1@cardiff.ac.uk

Kathryn Hughes[4], hugheska6@cardiff.ac.uk

Fiona V. Lugg-Widger[3], luggfv@cardiff.ac.uk

Rebecca Cannings-John[3], canningsrl@Cardiff.ac.uk

Fatemeh Torabi[2], fatemeh.torabi@swansea.ac.uk

Mandy Wootton[5], mandy.wootton@wales.nhs.uk

Ashley Akbari[2], a.akbari@swansea.ac.uk

Haroon Ahmed[1], ahmedh2@cardiff.ac.uk

Corresponding author

Nicola F. Reeve

Division of Population Medicine

Cardiff University

3rd Floor, Neuadd Meirionnydd

Heath Park

Cardiff

CF14 4YS

Reeven1@cardiff.ac.uk

+44(0)29 20687895

Affiliations

[1] Division of Population Medicine, Cardiff University, Cardiff, UK

[2] Population Data Science, Swansea University Medical School, Swansea University, Swansea, UK

[3] Centre for Trials Research, Cardiff University, Cardiff, UK

For peer review only

For peer review only - http://bmjopen.bmj.com/site/about/guidelines.xhtml

[4] PRIME Centre Wales, Cardiff University School of Medicine, Division of Population Medicine, Cardiff, UK

[5] Specialist Antimicrobial Chemotherapy Unit, Public Health Wales, Microbiology Cardiff, University Hospital of Wales, Cardiff, UK

Keywords

Myocardial Infarction, Stroke, Urinary Tract Infection

Word Count

3,383

For peer review only

For peer review only - http://bmjopen.bmj.com/site/about/guidelines.xhtml

Abstract

Introduction There is increasing interest in the relationship between acute infections and acute cardiovascular events. Most previous research has focussed on understanding whether the risk of acute cardiovascular events increases following a respiratory tract infection (RTI). The relationship between Urinary Tract Infections (UTIs) and acute cardiovascular events is less well studied. Therefore, the aim of this study is to determine whether there is a causal relationship between UTI and acute Myocardial Infarction (MI) or stroke.

Methods and analysis We will undertake a self-controlled case series study using linked anonymised general practice, hospital admission, and microbiology data held within the Secure Anonymised Information Linkage (SAIL) Databank. Self-controlled case series is a relatively novel study design where individuals act as their own controls, thereby inherently controlling for time-invariant confounders. Only individuals who experience an exposure and outcome of interest are included.

We will identify individuals in the SAIL Databank who have a hospital admission record for acute MI or stroke during the study period of 2010-2020. Individuals will need to be aged 30-100 during the study period and be Welsh residents for inclusion. UTI will be identified using general practice, microbiology, and hospital admissions data. We will calculate the incidence of MI and stroke in pre-defined risk periods following a UTI and in "baseline" periods (without UTI exposure) and use conditional Poisson regression models to derive incidence rate ratios.

Ethics and dissemination Data access, research permissions and approvals have been obtained from the SAIL independent Information Governance Review Panel (IGRP), project number 0972. Findings will be disseminated through conferences, blogs, social media threads and peer-reviewed journals. Results will be of interest internationally to primary and secondary care clinicians who manage UTIs and may inform future clinical trials of preventative therapy.

Article Summary

Strengths and limitations of this study

- The self-controlled case series method controls for time-invariant confounding, enabling more reliable causal estimates of the association between UTI and acute MI or stroke, compared to between-individual study designs.
- A causal relationship between UTI and acute MI or stroke has implications for our understanding of cardiovascular disease mechanisms and may inform new methods of disease prevention.
- Using individual-level population-scale anonymised, routinely collected electronic health record (EHR) data provides adequate power to study sub-groups and maximises representativeness and generalisability.
- EHR data are collected and recorded for clinical purposes, and therefore the reliability of research findings is dependent on the quality and completeness of these data.
- Clinical and microbiological diagnoses of UTI are subject to caveats, and therefore we will use several definitions of UTI that utilise the different data sources in the SAIL Databank.

For peer review only - http://bmjopen.bmj.com/site/about/guidelines.xhtml

1 INTRODUCTION

Since the late 1990s, an increasing number of observational studies have found an association between acute infections and myocardial infarction (MI).[1-10] Most studies focused on respiratory tract infections (RTIs), and found an increased risk of acute MI in the 1-3 days following an RTI, with the effect size varying according to the infecting organism.[2–8] For example, Kwong et al. found a six-fold increase in the risk of MI in the week after influenza infection, a four-fold increase after respiratory syncytial virus and a three-fold increase after other respiratory viruses.[5] Several studies have also found evidence of an association between pneumonia and acute cardiovascular events (including MI and stroke).[9–13] The increased risk of acute cardiovascular events following pneumonia infection persists for up to 10 years.[9] This long-term risk of acute cardiovascular events has also been observed after other severe infections, including sepsis and bacteraemia.[14–17]

It is thought that acute infection may cause major cardiovascular events through three mechanisms. First, the inflammatory response from acute infection may destabilise atherosclerotic plaques. Second, the prothrombotic, procoagulant state associated with acute infection may increase the risk of thrombosis at the site of plaque disruption. Third, inflammation and fever lead to an increase in heart rate, which may cause "demand ischemia" if the metabolic demands of the myocardial cells exceed oxygen supply.[1]

Urinary tract infections (UTI) can affect any part of the urinary system, including the kidneys, ureters, bladder and urethra. Most infections involve the lower urinary tract: the bladder and the urethra. UTIs are common infections, with 37% of women reporting experiencing at least one in their lifetime, and 29% experiencing more than one.[18] UTIs are associated with considerable morbidity. The global burden of disease study 2010 estimates the disability-adjusted life years attributable to tubulointerstitial nephritis, pyelonephritis and UTI to be 45 (95% uncertainty interval 32-55) per 100,000 population.[19]

The relationship between UTIs and acute cardiovascular events is less well studied than for RTIs. Only one previous study has examined this relationship. Smeeth et al. used the self-controlled case series method to analyse data from the General Practice Research Database. They found increased rates of MI and stroke subsequent to UTI, with the risk being highest in the first three days.[6] However, the data analysed are almost 20 years old, and there have been no attempts to replicate the findings. Furthermore, the study defined UTI using clinical codes only, so it is unclear if the reported associations related to individuals with clinical symptoms alone, or symptoms and bacteriuria, making it difficult to interpret whether individuals had true UTIs, or whether non-specific symptoms were misdiagnosed as UTI but represented early signs of a cardiovascular event. In addition, other studies have found that roughly two-thirds of women suspected to have UTI on presentation to primary care have no evidence of UTI on microbiological culture.[20] Therefore, the use of clinical codes alone to define UTI may lead to bias from misclassification of the exposure.

Therefore, the aim of this study is to determine whether there is a causal relationship between UTI and acute MI or stroke by analysing linked general practice, hospital admission, and microbiology data, from a representative sample of the Welsh population. We will use the self-controlled case series method, which controls for time-invariant confounding, enabling us to more reliably draw

For peer review only - http://bmjopen.bmj.com/site/about/guidelines.xhtml

causal inferences between UTI and acute MI or stroke, compared to between-individual study designs.

2 METHODS AND ANALYSIS

2.1 Aims and Objectives

The specific objectives of this research are to:

1) Estimate incidence rate ratios for acute MI and stroke in the 90 days following a clinically suspected and microbiologically confirmed UTI compared to baseline (all times outside of the 90-day risk period).
2) Assess the effect of different methods of UTI ascertainment on estimated rate ratios (i.e. clinically suspected and microbiologically confirmed; clinically diagnosed only; clinically suspected but not supported by microbiology; and UTI diagnosed and treated in hospital).
3) Investigate whether the effect of a clinically suspected and microbiologically confirmed UTI on acute MI and stroke differs according to the infecting organism.

Our primary hypothesis is that a clinically suspected and microbiologically confirmed UTI will increase the risk of acute MI or stroke in the 0-90-day post-infection period.

2.2 Data

We will use the Secure Anonymised Information Linkage (SAIL) Databank. This is an internationally recognised trusted research environment (TRE), with robust secure storage, enabling access to anonymised, linkable, individual-level Welsh population-scale data for research, with a focus on improving population health and health services. Data within SAIL is pseudonymised and made available to approved projects and users following an application to, and approval from the independent information governance review panel (IGRP). SAIL's storage and linkage processes ensure anonymity: first, data sources being provided to SAIL are split as per the standard split file process, with the source organisation splitting the source data into demographic data and clinical data, with a system linkage field to allow data to be re-joined later. This addresses confidentiality and disclosure issues that arise when working with health data by separating easily recognised person-based variables such as name and date of birth from clinical data, including information on diagnoses, tests and prescriptions. The demographic data are anonymised and assigned an Anonymised Linkage Field (ALF). These split files are then joined together using the system linkage field by the SAIL team and made available to researchers following encryption.[21-23]

We will use the SAIL Databank to access the following linked data: Welsh Longitudinal General Practice data (WLGP), Patient Episode Database for Wales (PEDW), Welsh Results Reporting Service (WRRS). The WLGP contains data from 84% of general practices in Wales, consisting of longitudinal data for 2.6 million Welsh residents, representing 84% of the population.[24] Demographic data, clinical diagnoses and prescription data are included. The PEDW contains ICD-10 coded diagnoses for admissions to any Welsh hospital.[25] The WRRS contains data on all tests requested from primary and secondary care NHS Wales organisations processed and analysed in NHS Wales laboratories, including requests for urine microscopy, culture and antibiotic susceptibilities.[26] Data are available from the data sources at varying times based on when clinical information

For peer review only - http://bmjopen.bmj.com/site/about/guidelines.xhtml

systems began, with data quality improving over time. As such, for our study, based on our approvals and where the data sources have consistent coverage and quality, we will be using them between 1st January 2010 and 31st December 2020.

2.3 Study Design

Individuals who experience UTIs and individuals who do not can differ in unmeasured ways and hence could be sources of residual confounding. We will use the self-controlled case series (SCCS) design method to deal with this issue. The SCCS method is an epidemiological study design for which individuals act as their own control so that both measured and unmeasured characteristics that vary between individuals are completely controlled.[27] Only individuals who have experienced an outcome and exposure of interest are included. The SCCS method compares the incidence of an outcome during pre-defined risk periods with incidence during baseline periods (all times outside of risk and pre-risk periods) and estimates the temporal association between a transient exposure and outcome. Time-invariant covariates (e.g., sex) are inherently controlled for, and time varying covariates (e.g., age) are adjusted for within the models. The method was originally developed to investigate associations between vaccinations and acute adverse events, such as aseptic meningitis,[28, 29] but has since been applied in a range of epidemiological settings,[30–32] including non-acute events such as autism.[33] As with other study designs, the SCCS method makes several assumptions that need to be met in order to obtain valid and unbiased estimates, but there are model extensions which provide solutions to violations of these assumptions under certain circumstances.[34] The model assumptions, how they apply to our study, and the solutions to violations of the assumptions are given in Table 1.

A diagrammatic representation of observation time for an individual in the proposed SCCS design is given in Figure 1. Risk periods start on the date of a UTI and end 90 days later. The date of a UTI is the earliest date of occurrence of any of the events necessary for each different UTI definition. E.g., the definition of UTI in our primary analysis is a combination of a UTI-related diagnostic code and antibiotic prescription in WLGP data and a urine culture result that supports a diagnosis of UTI in the WRRS data, occurring within a 7-day window. In this case, the date of the UTI would be the date of whichever of the following events occurred first: UTI related diagnostic code, antibiotic prescription, supporting urine culture.

Individuals can have more than one UTI during the observation period, and therefore can have more than one 90-day risk period. Where risk periods overlap, the later period takes precedence, and the earlier period is shortened. There will be a pre-risk period of 7 days before the risk period, to allow for the situation where an individual has a UTI for several days prior to consultation, so events in the pre-risk period are not erroneously attributed to the baseline period.

Baseline periods are all times outside of the risk and pre-risk periods. The study period is from 1st January 2010 to 31st December 2020. The observation period is different for each individual. It generally follows the study period but may start later for some individuals who were not Welsh residents at the start of the study period but became so later, or who turned 30 years of age sometime during the study period. Similarly, the observation period may end before the study period when individuals moved out of Wales or died.

For peer review only - http://bmjopen.bmj.com/site/about/guidelines.xhtml

Table 1 Model assumptions and solutions to violations of those assumptions

Assumption	How the assumption applies to this study	Solution	Example of use of the solution in the literature
Subsequent exposures should not be affected by previous events.	We might see a temporary increase in UTIs subsequent to an MI or stroke event, which would bias estimates towards the null.	Apply a pre-risk period.	Gibson et al. studied the association between prescription drugs and road traffic accidents. As some drugs may be used to treat anxiety or pain caused by the crash, a 4-week pre-exposure period was included.[35]
	As both MI and stroke have relatively high death rates, the length of the observation period is dependent on events, and no further exposures are possible after death.	Use an event-dependent observation period model extension [36] and conduct a sensitivity analysis that repeats the analysis, excluding individuals who died within 30 days of the event.	Bruer et al. used the event-dependent observation period model extension in their study on the association between antipsychotic drugs and myocardial infarction.[37]<br>Langan et al. studied the risk of stroke following herpes zoster. They conducted a sensitivity analysis excluding individuals who died within 90 days of stroke.[38]
Event rates are constant within defined periods	MI and stroke are more common in older individuals and may be affected by seasonal changes.	Control for age and season effects.	Grave et al. studied the association between seasonal influenza vaccination and Guillan Barre syndrome. They adjusted for calendar month, as the vaccinations are seasonal by design.[39]<br>In a study of the association between chickenpox and stroke, Thomas et al. adjusted for age in 5-year age bands.[40]
Events are independently recurrent or rare.	MI and stroke are not independent: once an individual has a first event, they are more likely to have a second.	Study first events only.	Langan et al. began the observation period 12 months into follow-up time to ensure first stroke events had been correctly identified.[38]

For peer review only - http://bmjopen.bmj.com/site/about/guidelines.xhtml

2.4 Population

The SCCS method starts with identifying individuals who have had the outcome of interest. Therefore, the source population are individuals within the SAIL Databank who have a hospital admission record for acute MI or stroke during the study period. For inclusion, individuals will need to be aged 30-100 between 1st January 2010 and 31st December 2020 and be Welsh residents. We chose a lower age bound of 30 years to reduce the chance of including MIs and strokes due to congenital or other non-artherosclerotic causes. A lower age bound of 40 might reduce this chance further but would increase the chance of missing relevant events, especially given the greater burden of cardiovascular disease in Wales compared to the UK as a whole. [41]

2.5 Outcomes

Outcomes of interest are acute MI or stroke, as identified by International Classification of Disease version 10 (ICD-10) codes from inpatient diagnoses recorded in the Patient Episode Database for Wales. A list of ICD-10 codes to be used are given in Supplemental Material A.

2.6 Exposure

The exposure of interest is UTI. The risk period is 0-90 days following a UTI. This period was chosen as previous research has shown an increase in acute MI and stroke risk in the 1-90 days following a UTI.[6] There will be a pre-risk period of seven days before the risk period, to allow for the situation where an individual has a UTI for several days prior to consultation, so events in this period are not erroneously attributed to the baseline period. Individuals can be exposed to a UTI more than once during the observation period. Each exposure will be followed by the same 90-day risk period. Baseline periods are all other times.

To ascertain UTI, we developed definitions that reflected the Public Health Wales Microbiology Division's standard operating procedure for the investigation of urine.[42] These procedures are followed by NHS microbiology laboratories across Wales. For each definition, the data sources required and the clinical scenario represented is summarised in Table 2, and the code lists used are given in Supplemental Material B-D. In our primary analysis, an individual will be regarded as being exposed to a UTI if the following events occur within a 7-day window:

1. A GP record of a UTI diagnostic or symptom code
2. A GP record of an antibiotic prescription
3. A microbiology record of a urine sample with bacterial growth of a single organism of >=10^8 colony forming units (cfu) per litre and white blood cells >=10^8 per litre. If there are two organisms grown, both must demonstrate growth of >=10^8 cfu per litre. More than two organisms will be regarded as mixed growth and thus not supportive of a UTI diagnosis. In a sensitivity analysis, we will widen the microbiological criteria and include all urine samples with bacterial growth of a single organism of >=10^7 colony forming units (cfu) per litre, irrespective of the white blood cell count.

In secondary analysis 1, we will estimate the risk of MI and stroke amongst individuals with a GP record of a UTI code and antibiotic prescription, and a microbiology record of a urine sample with mixed bacterial growth (any descriptor for 'mixed growth' or >3 organisms). This is an important analysis given the uncertain clinical significance of mixed bacterial growth in an individual with symptoms of UTI. In secondary analysis 2, an individual will be regarded as exposed to UTI with only a GP record of a diagnostic or symptom code and an antibiotic prescription (no microbiology).

For peer review only - http://bmjopen.bmj.com/site/about/guidelines.xhtml

Secondary analysis 3 will estimate the risk of MI and stroke amongst individuals where UTI was suspected and treated by the GP, but urine microbiology showed bacterial growth of <10^7 cfu per litre (not supportive of a UTI diagnosis). Secondary analysis 4 will focus on individuals with a hospital admission with a UTI-related ICD-10 code and a microbiology record of a urine sample with bacterial growth of a single organism of >=10^8 cfu per litre and white blood cells >=10^8 per litre. As for the primary analysis, if there are two organisms grown, both must demonstrate growth of >=10^8 cfu per litre, and more than two organisms will be regarded as mixed growth. Secondary analysis 5 combines the primary analysis, and secondary analysis 4, considering individuals with either a GP record of a UTI code and antibiotic prescription, or a hospital admission with a UTI-related ICD-10 code, and a microbiology record of a urine sample with bacterial growth of a single organism of >=10^8 cfu per litre and white blood cells >=10^8 per litre. In the primary analysis, any hospital diagnosed UTI is likely to count towards baseline time, whereas they would be included in exposed time here.

Table 2 Definitions of UTI for primary and secondary analyses. As the UTI definitions are combinations of two or more components, the start of the risk period is defined as the date of the earliest component

	**UTI-related Read code in GP data (Supplemental Material B)**	**Antibiotic prescription in GP data (Supplemental Material C)**	**UTI-related ICD-10 code in PEDW (Supplemental Material D)**	**Urine culture results in WRRS**	**Time frame**	**Clinical scenario**
**Primary analysis**	Yes	Yes	No	Yes, showing bacterial growth of >=10^8 cfu/L and WBC>= 10^8/L	Three codes occur within a 7-day window	GP clinically suspected and microbiologically confirmed UTI
**Secondary analysis 1**	Yes	Yes	No	Yes, showing mixed bacterial growth (any descriptor for 'mixed growth' or >3 organisms).	Three codes occur within a 7-day window	GP clinically suspected UTI with mixed growth

For peer review only - http://bmjopen.bmj.com/site/about/guidelines.xhtml

**Secondary analysis 2**	Yes	Yes	No	No	Same day	GP clinically diagnosed and treated UTI. It is important to consider this group as not all individuals with suspected UTI have urine culture, and limiting to those with culture is subject to selection bias.
**Secondary analysis 3**	Yes	Yes	No	Yes, showing bacterial growth of < 10^7 cfu per litre	Three codes occur within a 7-day window	UTI is clinically suspected but not supported by microbiology. This group is important to understand if early symptoms and signs of acute MI or stroke are attributed to UTI.
**Secondary analysis 4**	No	No	Yes	Yes, showing bacterial growth of $>=10^8$ cfu/L and WBC $>=10^8$/L	Two codes occur within a 7-day window	UTI diagnosed and/or treated in hospital
**Secondary analysis 5**	Yes, OR ICD-10 code	Yes, OR ICD-10 code	Yes, OR: UTI Read code AND antibiotic Read code	Yes, showing bacterial growth of $>=10^8$ cfu/L and WBC $>=10^8$/L	Two/three codes occur within a 7-day window	GP clinically suspected and microbiologically confirmed UTI or UTI diagnosed and/or treated in hospital

For peer review only

2.7 Statistical Analysis

We will describe the demographics of the study population, such as age and sex distribution, medical history, and prescribed medication, using means and standard deviations for continuous variables, and frequencies and proportions for categorical variables. For each analysis, we will use conditional logistic regression to estimate incidence rate ratios (IRRs), with 95% confidence intervals, for the risk of acute MI or stroke in pre-risk and risk periods compared to baseline periods. We will include only individuals who have experienced both the outcome and the exposure, and will include only the first acute MI or stroke diagnosis in the observation period. The IRRs will be adjusted for time-varying confounders: age, season and year of UTI diagnosis. Year of UTI diagnosis is included because diagnostic and coding practices may have changed over time as a result of guidance and

For peer review only - http://bmjopen.bmj.com/site/about/guidelines.xhtml

awareness around microbial resistance. Adjusted IRRs will be reported for the risk of acute MI or stroke at 1-7, 8-14, 15-28 and 29-90 days after UTI. We will conduct the analysis in R, using the SCCS package. We will report the findings in accordance with RECORD and STROBE.

2.8 Sensitivity and sub-group analyses

We will perform several sensitivity and sub-group analyses to assess the robustness of the findings of our primary analysis to different assumptions:

- We will explore the impact of using a wider definition of MI and stroke, including ICD-10 codes for acute coronary syndromes and transient ischaemic attacks. This will include events that have potentially been missed by our main definition and assess how sensitive our findings are to the definition of MI and stroke.
- We will explore the impact of widening the microbiological definition of UTI to bacterial growth of a single organism of >=10^7 cfu per litre irrespective of WBC count.
- We will differentiate first-ever MI or stroke from recurrent events, and report risk estimates separately. This analysis will exclude individuals with a PEDW record of an event before the observation period, and include only those who have their first ever event during the observation period.
- We will extend the pre-risk period to 14 days. Some individuals may have had a UTI for several days prior to diagnosis, and so an acute MI or stroke during this time may relate to exposure but without a pre-risk period, would count towards the baseline period.
- We will repeat the analysis excluding individuals who died within 30 days of an event to examine the potential effect of an event dependent observation period.
- We will restrict the definition of UTI to include only antibiotic prescriptions for nitrofurantoin (currently recommended 1st line therapy) to explore whether the choice of antibiotic impacts the findings.
- We will use interaction terms to assess the impact of specific bacterial organisms on the relationship between UTI and MI or stroke.
- We will examine whether the COVID-19 pandemic may affect our findings by (1) excluding individuals whose MI or stroke occurred in 2020, and (2) including an interaction term to explore whether the association between exposure and outcome differs in 2020 versus pre-2020.
- We will include an interaction term to explore whether the association between exposure and outcome differs in those with and without a history of diabetes, given its potential role as a risk factor of both UTI and MI/stroke.

2.9 Sample Size and Power

Our initial work has identified 51,656 individuals with acute MI and 58,146 with stroke in the SAIL Databank who meet all inclusion criteria. In the previous study by Smeeth et al.,[6] the sample size was 53,709 for acute MI, and 55,157 for stroke, where 16% of acute MI cases, and 21% of stroke cases were exposed to UTI.[6] Based on a conservative exposure rate of 10%, and an at-risk window of 90 days, we estimated the effect size that could be reliably detected with our potential sample size, using the sample size function in the SCCS package in R. The available sample provides 90% power to detect an incidence rate ratio (IRR) of 1.3 at the alpha = 0.05 level, which is smaller than the IRRs detected in Smeeth et al.[6]

For peer review only - http://bmjopen.bmj.com/site/about/guidelines.xhtml

2.10 Patient and Public Involvement

We developed this research proposal in collaboration with members of the Wales Centre for Primary and Emergency Care Research Service Users group (SUPER). We have a PPI plan and are consulting the SAIL consumer panel and SUPER regarding all stages of this research, including ongoing discussion of analysis plans, review of findings, and plans for dissemination (e.g. public facing outputs). We have extended our sub-group analysis to include individuals with diabetes in response to discussions with the SAIL consumer panel members. Identifying patient and public involvement for future stages of this research is a secondary objective of our PPI plan.

2.11 Summary of Cases

The individuals with an ICD-10 code for either MI or stroke were selected according to the eligibility criteria, as shown in Figure 2. There are 58,146 individuals with an ICD-10 code for stroke, and 51,656 individuals with an ICD-10 code for MI, a total of 105,930 unique individuals (it is possible for an individual to be in both the stroke and the MI group). Stroke cases were 49% male, MI cases 63% male. Female stroke cases were older than males, with a median age of 79 years (25th to 75th centiles 69-87) compared to a median of 74 years (25th to 75th centiles 64-82) for males. Female MI cases were also older, with a median age of 77 (25th to 75th centiles 66-85) compared to 69 (25th to 75th centiles 59-78) for males.

The mean length of observation (study entry to study exit) of stroke cases was 2,796 days for females and 2,981 days for males. For MI cases, 2,981 for females and 3,251 for males. History of diagnoses and prescription drugs is taken from all health records available for each individual. Health data is available for a median of 13 years (25th to 75th centiles 8-16) prior to the study start.

Characteristics of cases, including a history of diagnoses and prescription drugs prior to an event, smoking status, Welsh Index of Multiple Deprivation (WIMD) version 2019 and electronic Frailty Index (eFI) are given in Table 3.

For peer review only - http://bmjopen.bmj.com/site/about/guidelines.xhtml

Table 3 Characteristics of cases of stroke and MI

Characteristic	Stroke	MI
Sex (% male)	49.4	62.8
Age of males (mean (25th, 75th centiles))	74 (64, 82)	69 (59, 78)
Age of females (mean (25th, 75th centiles))	79 (69, 87)	77 (66, 85)
Welsh Index of Multiple Deprivation (WIMD) 2019 quintiles*		
1 (least deprived)	19.8	20.6
2	20.8	20.5
3	20.3	20.9
4	20.4	20.1
5 (most deprived)	18.6	17.9
Current smoker (%)	19.8	24.2
Electronic frailty index (mean (sd))	0.16 (0.12)	0.15 (0.12)
Prescribed lipid lowering drugs (%)	49.0	49.6
Prescribed aspirin (%)	43.7	41.3
Prescribed hypertensive drugs (%)	59.1	55.1
Prescribed beta blockers (%)	39.6	37.3
Chronic Kidney Disease (%)	20.9	18.9
COPD (%)	9.9	10.9
Asthma (%)	15.1	16.0
Hypertension (%)	51.2	46.2
Diabetes (%)	19.4	20.7
Cardiovascular disease (%)	56.4	53.4
Coronary Heart Disease (%)	15.9	21.5
Atrial Fibrillation (%)	16.0	8.7
Heart Failure (%)	8.4	8.6
Peripheral Vascular Disease (%)	7.2	7.7
Angina (%)	11.3	16.0
Transient Ischaemic Attacks (%)	9.4	5.0
Total number of cases	58,150	51,659

* LSOA version 2011 and WIMD version 2019

2.12 Ethics and Dissemination

All study data will be held within the SAIL Databank, an ISO27001 certified trusted research environment (TRE) for anonymised individual-level data. Data access, research permissions and approvals have been obtained from the SAIL independent Information Governance Review Panel (IGRP), project number 0972. Analyses will be conducted within the SAIL TRE. There are strict disclosure control processes in place. Only aggregated outputs will be approved for release to ensure individuals are not identified. Findings will be disseminated through peer-review journals and conferences. Results will be of interest internationally to primary and secondary care clinicians who manage UTIs. An association between UTI and either MI or stroke will support a future funding application for a randomised trial of preventative treatments, such as antiplatelet drugs.

For peer review only

3 ACKNOWLEDGMENTS

This study makes use of anonymised data held in the Secure Anonymised Information Linkage (SAIL) Databank. We would like to acknowledge all the data providers who make anonymised data available for research.

4 AUTHOR CONTRIBUTIONS

HA is the chief investigator of the study. All authors have contributed to and are responsible for the final design of the study. NFR is responsible for study management. NFR, VB, FT and AA are responsible for the data management. NFR and RC-J are responsible for statistical planning and analysis. FLW is leading the patient and public involvement plans for the study. HA and MW are responsible for the microbiological definition of UTI. All authors have read and approved the final manuscript (NFR, VB, DG, KH, FLW, RC-J, FT, MW, AA, HA).

5 FUNDING STATEMENT

This work was supported by The British Heart Foundation, grant number PG/20/10419. The Centre for Trials Research is funded by Health and Care Research Wales (grant number N/A) and Cancer Research UK (grant number N/A). Population Data Science, Swansea University is supported by Health Data Research UK (HDR-9006) and ADR Wales (grant ES/S007393/1). PRIME Centre Wales is funded by Health and Care Research Wales.

6 COMPETING INTERESTS STATEMENT

None declared

REFERENCES

[1] Musher DM, Abers MS, Corrales-Medina VF. Acute infection and myocardial infarction. *NEJM.* 2019;380(2):171-6.

[2] Warren-Gash C, Smeeth L, Hayward AC. Influenza as a trigger for acute myocardial infarction or death from cardiovascular disease: a systematic review. *Lancet Infect Dis*. 2009;9(10):601-10.

[3] Warren-Gash C, Hayward AC, Hemingway H, et al. Influenza infection and risk of acute myocardial infarction in England and 9 Wales: a CALIBER self-controlled case series study. *The Journal of infectious diseases.* 2012;206(11):1652-9.

[4] Warren-Gash C, Blackburn R, Whitaker H, et al. Laboratory-confirmed respiratory infections as triggers for acute myocardial infarction and stroke: a self-controlled case series analysis of national linked datasets from Scotland. *Eur Respir J*. 2018;51(3).

[5] Kwong JC, Schwartz KL, Campitelli MA, et al. Acute myocardial infarction after laboratory-confirmed influenza infection. *NEJM*. 2018;378(4):345-53.

For peer review only - http://bmjopen.bmj.com/site/about/guidelines.xhtml

[6] Smeeth L, Thomas SL, Hall AJ, et al. Risk of myocardial infarction and stroke after acute infection or vaccination. *NEJM*. 2004;351(25):2611-8.

[7] Clayton TC, Thompson M, Meade TW. Recent respiratory infection and risk of cardiovascular disease: case-control study through a general practice database. *Eur Heart J*. 2008;29(1):96-103.

[8] Warren-Gash C, Geretti AM, Hamilton G, et al. Influenza-like illness in acute myocardial infarction patients during the winter wave of the influenza A H1N1 pandemic in London: a case–control study. *BMJ Open*. 2013;3(5):e002604.

[9] Corrales-Medina VF, Alvarez KN, Weissfeld LA, et al. Association between hospitalization for pneumonia and subsequent risk of cardiovascular disease. *JAMA*. 2015;313(3):264-74.

[10] Corrales-Medina VF, Madjid M, Musher DM. Role of acute infection in triggering acute coronary syndromes. *Lancet Infect Dis*. 2010;10(2):83-92.

[11] Musher DM, Rueda AM, Kaka AS, et al. The association between pneumococcal pneumonia and acute cardiac events. *Clin Infect Dis*. 2007;45(2):158-65.

[12] Corrales-Medina VF, Serpa J, Rueda AM, et al. Acute bacterial pneumonia is associated with the occurrence of acute coronary syndromes. *Medicine.* 2009;88(3):154-9.

[13] Violi F, Cangemi R, Falcone M, et al. Cardiovascular complications and short-term mortality risk in community-acquired pneumonia. *Clin Infect Dis*. 2017;64(11):1486-93.

[14] Dalager-Pedersen M, Søgaard M, Schønheyder HC, et al. Risk for myocardial infarction and stroke after community-acquired bacteremia: a 20-year population-based cohort study. *Circulation*. 2014;129(13):1387-96. 10

[15] Bergh C, Fall K, Udumyan R, et al. Severe infections and subsequent delayed cardiovascular disease. *Eur J Prev Cardiol*. 2017;24(18):1958-66.

[16] Ou SM, Chu H, Chao PW, et al. Long-term mortality and major adverse cardiovascular events in sepsis survivors. A nationwide population-based study. *Am J Respir Crit Care Med.* 2016;194(2):209-17.

[17] Jafarzadeh SR, Thomas BS, Warren DK, et al. Longitudinal study of the effects of bacteremia and sepsis on 5-year risk of cardiovascular events. *Rev Infect Dis*. 2016;63(4):495-500.

[18] Butler CC, Hawking MK, Quigley A, et al. Incidence, severity, help seeking, and management of uncomplicated urinary tract infection: a population-based survey. *Br J Gen Pract*. 2015;65(639):e702-7.

[19] Murray CJ, Vos T, Lozano R, et al. Disability-adjusted life years (DALYs) for 291 diseases and injuries in 21 regions, 1990–2010: a systematic analysis for the Global Burden of Disease Study 2010. *Lancet*. 2012;380(9859):2197-223.

For peer review only - http://bmjopen.bmj.com/site/about/guidelines.xhtml

[20] Butler CC, Francis N, Thomas-Jones E, et al. Variations in presentation, management, and patient outcomes of urinary tract infection: a prospective four-country primary care observational cohort study. *Br J Gen Pract*. 2017;67(665):e830-41.

[21] Jones KH, Ford DV, Thompson S, et al. A profile of the SAIL databank on the UK secure research platform. *Int J Popul Data Sci.* 2019;4(2).

[22] Lyons RA, Jones KH, John G, et al. The SAIL databank: linking multiple health and social care datasets. *BMC Med Inform Decis Mak*. 2009;9(1):1-8.

[23] Ford DV, Jones KH, Verplancke JP, et al. The SAIL Databank: building a national architecture for e-health research and evaluation. *BMC Health Serv Res.* 2009;9(1):1-12.

[24] SAIL. Welsh Longitudinal GP Dataset - Welsh Primary Care (WLGP); 2021. Available from: https://web.www.healthdatagateway.org/dataset/ 33fc3ffd-aa4c-4a16-a32f-0c900aaea3d2.

[25] SAIL. Patient Episode Dataset for Wales (PEDW); 2021. Available from: https://web.www.healthdatagateway.org/dataset/ 4c33a5d2-164c-41d7-9797-dc2b008cc852.

[26] SAIL. Welsh Results Reports Service (WRRS); 2021. Available from: https://web. www.healthdatagateway.org/dataset/71d37610-ac55-432d-82a3-bdb04407acd8.

[27] Farrington P, Whitaker H, Weldeselassie YG. Self-controlled case series studies: a modelling guide with R. Chapman and Hall/CRC; 2018.

[28] Miller E, Farrington P, Goldracre M, et al. Risk of aseptic meningitis after measles, mumps, and rubella vaccine in UK children. *Lancet.* 1993;341(8851):979-82.

[29] Farrington P, Rush M, Miller E, et al. A new method for active surveillance of adverse events from diphtheria/tetanus/pertussis and measles/mumps/rubella vaccines. *Lancet.* 1995;345(8949):567-9.

[30] Whitaker HJ, Paddy Farrington C, Spiessens B, et al. Tutorial in biostatistics: the self-controlled case series method. *Stat Med.* 2006;25(10):1768-97.

[31] Douglas IJ, Smeeth L. Exposure to antipsychotics and risk of stroke: self controlled case series study. *BMJ*. 2008;337.

[32] Douglas IJ, Evans SJ, Pocock S, et al. The risk of fractures associated with thiazolidinediones: a self-controlled case-series study. *PLoS Med*. 2009;6(9):e1000154.

[33] Taylor B, Miller E, Farrington C, et al. Autism and measles, mumps, and rubella vaccine: no epidemiological evidence for a causal association. *Lancet*. 1999;353(9169):2026-9.

[34] Petersen I, Douglas I, Whitaker H. Self controlled case series methods: an alternative to standard epidemiological study designs. *BMJ.* 2016;354.

[35] Gibson JE, Hubbard RB, Smith CJ, et al. Use of self-controlled analytical techniques to assess

For peer review only - http://bmjopen.bmj.com/site/about/guidelines.xhtml

the association between use of prescription medications and the risk of motor vehicle crashes. *Am J Epidemiol.* 2009;169(6):761-8.

[36] Farrington CP, Anaya-Izquierdo K, Whitaker HJ, et al. Self-controlled case series analysis with event-dependent observation periods. *J Am Stat Assoc*. 2011;106(494):417-26.

[37] Brauer R, Smeeth L, Anaya-Izquierdo K, et al. Antipsychotic drugs and risks of myocardial infarction: a self-controlled case series study. *Eur Heart J*. 2015;36(16):984-92.

[38] Langan SM, Minassian C, Smeeth L, et al. Risk of stroke following herpes zoster: a self-controlled case-series study. *Clin Infect Dis*. 2014;58(11):1497-503.

[39] Grave C, Boucheron P, Rudant J, et al. Seasonal influenza vaccine and Guillain-Barré syndrome: a self-controlled case series study. *Neurology*. 2020;94(20):e2168-79. 12

[40] Thomas SL, Minassian C, Ganesan V, et al. Chickenpox and risk of stroke: a self-controlled case series analysis. *Clin Infect Dis*. 2014;58(1):61-8.

[41] Steel N, Ford JA, Newton JN, et al. Changes in health in the countries of the UK and 150 local authority areas 1990-2016: a systematic analysis for the Global Burden of Disease Study 2016. *Lancet*. 2018; 392(10158):1647-1661.

[42] Microbiology Division Public Health Wales. CDBBA 041 Bench Aid: Investigation of Urine; 2021.

For peer review only

For peer review only - http://bmjopen.bmj.com/site/about/guidelines.xhtml

FIGURE LEGENDS

Figure 1 Diagrammatic representation of observation time for an individual in the proposed Self-Controlled Case Series design

Figure 2: Case selection process. *Individuals appear and are counted in both the stroke and MI datasets if they had both an MI and a stroke event within the study period.

For peer review only

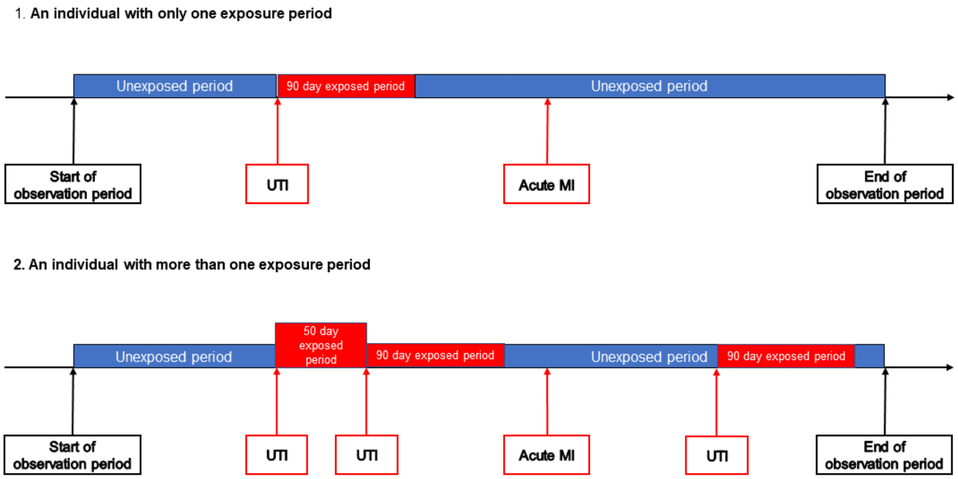

Diagrammatic representation of observation time for an individual in the proposed Self-Controlled Case Series design

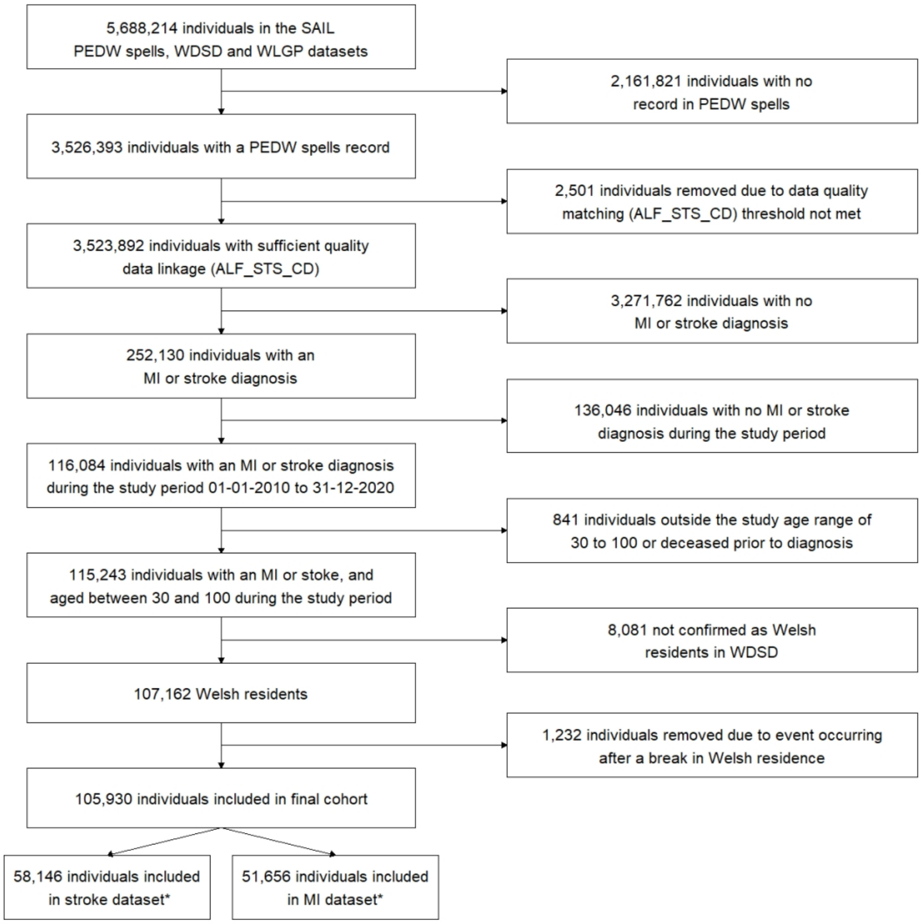

Case selection process. *Individuals appear and are counted in both the stroke and MI datasets if they had both an MI and a stroke event within the study period.

Myocardial Infarction and stroke subsequent to urinary tract infection (MISSOURI): protocol for a self-controlled case series using linked electronic health records: Supplemental Material

Supplemental Material A - ICD-10 Codes for Acute MI and Stroke to Identify Potential Cases

Code	Description	Condition
I210	Acute transmural myocardial infarction of anterior wall	MI
I211	Acute transmural myocardial infarction of inferior wall	MI
I212	Acute transmural myocardial infarction of other sites	MI
I213	Acute transmural myocardial infarction of unspecified site	MI
I214	Acute subendocardial myocardial infarction	MI
I219	Acute myocardial infarction unspecified	MI
I220	Subsequent myocardial infarction of anterior wall	MI
I221	Subsequent myocardial infarction of inferior wall	MI
I228	Subsequent myocardial infarction of other sites	MI
I229	Subsequent myocardial infarction of unspecified site	MI
I600	Subarachnoid haemorrhage from carotid siphon and bifurcation	STROKE
I601	Subarachnoid haemorrhage from middle cerebral artery	STROKE
I602	Subarachnoid haemorrhage from anterior communicating artery	STROKE
I603	Subarachnoid haemorrhage from posterior communicating artery	STROKE
I604	Subarachnoid haemorrhage from basilar artery	STROKE
I605	Subarachnoid haemorrhage from vertebral artery	STROKE
I606	Subarachnoid haemorrhage from other intracranial arteries	STROKE
I607	Subarachnoid haemorrhage from intracranial artery unspec	STROKE
I608	Other subarachnoid haemorrhage	STROKE
I609	Subarachnoid haemorrhage unspecified	STROKE
I610	Intracerebral haemorrhage in hemisphere subcortical	STROKE
I611	Intracerebral haemorrhage in hemisphere cortical	STROKE
I612	Intracerebral haemorrhage in hemisphere unspecified	STROKE
I613	Intracerebral haemorrhage in brain stem	STROKE
I614	Intracerebral haemorrhage in cerebellum	STROKE
I615	Intracerebral haemorrhage intraventricular	STROKE
I616	Intracerebral haemorrhage multiple localized	STROKE
I618	Other intracerebral haemorrhage	STROKE
I619	Intracerebral haemorrhage unspecified	STROKE
I629	Intracranial haemorrhage (nontraumatic)unspecified	STROKE
I630	Cerebral infarct due to thrombosis of precerebral arteries	STROKE
I631	Cerebral infarction due to embolism of precerebral arteries	STROKE
I632	Cereb infarct due unsp occlusion or stenos precerebrl arts	STROKE
I633	Cerebral infarction due to thrombosis of cerebral arteries	STROKE
I634	Cerebral infarction due to embolism of cerebral arteries	STROKE
I635	Cerebrl infarct due unspec occlusion or stenos cerebrl arts	STROKE
I636	Cerebral infarction due to cerebral venous thrombosis, nonpyogenic	
I638	Other cerebral infarction	STROKE
I639	Cerebral infarction unspecified	STROKE
I64X	Stroke not specified as haemorrhage or infarction	STROKE

For peer review only

Supplemental Material B - Read Codes for UTI to Determine Risk Periods

Code	Description
1A1..	Urinary frequency/Frequency of micturition/Micturition frequency/Polyuria
1A12.	Frequency of micturition
1A1Z.	Micturition frequency NOS
1A44.	Urine looks cloudy
1A45.	Blood in urine - haematuria/Blood in urine - symptom/Haematuria - symptom
1A55.	Dysuria
1AG..	Recurrent urinary tract infections
1AZ6.	Lower urinary tract symptoms
1J4..	Suspected UTI
K0A2.	Recurrent and persistent haematuria
K101.	Acute pyelonephritis
K101z	Acute pyelonephritis NOS
K10y0	Pyelonephritis unspecified
K15..	Cystitis
K150.	Acute cystitis
K152.	Other chronic cystitis
K152y	Chronic cystitis unspecified
K152z	Other chronic cystitis NOS
K155.	Recurrent cystitis
K15yz	Other cystitis NOS
K15z.	Cystitis NOS
K190.	Recurrent urinary tract infection/Urinary tract infection, site not specified
K1903	Recurrent UTI/Recurrent urinary tract infection
K1905	Urinary tract infection
K190z	Urinary tract infection, site not specified NOS
K1970	Painless haematuria
K1971	Painful haematuria
K1973	Frank haematuria
Kyu51	[X]Other cystitis
L1668	Urinary tract infection complicating pregnancy
R08..	[D]Urinary system symptoms
R081.	[D]Dysuria
R081z	[D]Dysuria NOS
R084.	[D]Micturition frequency and polyuria
R0840	[D]Frequency of micturition, unspecified
R0842	[D]Nocturia
R084z	[D]Frequency of micturition or polyuria NOS
R0908	[D]Suprapubic pain
SP07Q	Catheter-associated urinary tract infection/CAUTI - catheter-associated urinary tract infection

For peer review only

Supplemental Material C - Read Codes for Antibiotics to Determine Risk Periods

Code	Description
e31B.	*AMIX 250mg/5mL suspension
e319.	*AMOXIL SF 125mg/5mL syrup
e31a.	*AMOXIL SF 250mg/5mL syrup
e31d.	*AMOXIL SF 3g sachets
e31R.	*AMOXYMED 250mg capsules
e31i.	*AUGMENTIN 375mg disp tablets
e69e.	*CEPOREX 250mg/5mL suspension
eg68.	*CIPROXIN 100mg tablets
e31v.	*CO-AMOXICLAV 125mg/5mL susp
e31u.	*CO-AMOXICLAV 375mg disp tabs
egA3.	*FOSFOMYCIN 3g/sachet granules
eg17.	*MACRODANTIN 100mg capsules
eg16.	*MACRODANTIN 50mg capsules
eccb.	*MONOTRIM 50mg/5mL s/f susp
egA1.	*MONURIL 3g/sach granules
ecc3.	*TRIMETHOPRIM 300mg tablets
e3z5.	AMIX 250mg capsules
e3z6.	AMIX 500mg capsules
e3zo.	AMOXICILLIN 125mg/1.25mL susp
e3zk.	AMOXICILLIN 125mg/5mL s/f susp
e3zm.	AMOXICILLIN 125mg/5mL syrup
e311.	AMOXICILLIN 250mg capsules
e3zu.	AMOXICILLIN 250mg/5mL s/f susp
e3zn.	AMOXICILLIN 250mg/5mL syrup
e312.	AMOXICILLIN 500mg capsules
e3zq.	AMOXICILLIN powder 3g/sachet
e31b.	AMOXIL 125mg/1.25mL paed susp
e315.	AMOXIL 250mg capsules
e316.	AMOXIL 500mg capsules
e3zo.	AMOXYCILLIN 125mg/1.25mL susp
e3zk.	AMOXYCILLIN 125mg/5mL s/f susp
e3zm.	AMOXYCILLIN 125mg/5mL syrup
e311.	AMOXYCILLIN 250mg capsules
e3zu.	AMOXYCILLIN 250mg/5mL s/f susp
e3zn.	AMOXYCILLIN 250mg/5mL syrup
e312.	AMOXYCILLIN 500mg capsules
e3zq.	AMOXYCILLIN powder 3g/sachet
e31k.	AUGMENTIN 125/31 in 5mL susp
e31P.	AUGMENTIN 250/62 in 5mL susp
e31h.	AUGMENTIN 375mg tablets
e31T.	AUGMENTIN 625mg tablets
e31Y.	AUGMENTIN-DUO 400/57in5mL susp
e61C.	CEFACLOR 125mg/5mL s/f susp
e615.	CEFACLOR 125mg/5mL suspension
e614.	CEFACLOR 250mg capsules
e61D.	CEFACLOR 250mg/5mL s/f susp
e616.	CEFACLOR 250mg/5mL suspension
e61a.	CEFACLOR 375mg m/r tablets
e618.	CEFACLOR 500mg capsules

Code	Description
e31B.	*AMIX 250mg/5mL suspension
e319.	*AMOXIL SF 125mg/5mL syrup
e31a.	*AMOXIL SF 250mg/5mL syrup
e31d.	*AMOXIL SF 3g sachets

For peer review only

e31R.	*AMOXYMED 250mg capsules
e31i.	*AUGMENTIN 375mg disp tablets
e69e.	*CEPOREX 250mg/5mL suspension
eg68.	*CIPROXIN 100mg tablets
e31v.	*CO-AMOXICLAV 125mg/5mL susp
e31u.	*CO-AMOXICLAV 375mg disp tabs
egA3.	*FOSFOMYCIN 3g/sachet granules
eg17.	*MACRODANTIN 100mg capsules
eg16.	*MACRODANTIN 50mg capsules
eccb.	*MONOTRIM 50mg/5mL s/f susp
egA1.	*MONURIL 3g/sach granules
ecc3.	*TRIMETHOPRIM 300mg tablets
e3z5.	AMIX 250mg capsules
e3z6.	AMIX 500mg capsules
e3zo.	AMOXICILLIN 125mg/1.25mL susp
e3zk.	AMOXICILLIN 125mg/5mL s/f susp
e3zm.	AMOXICILLIN 125mg/5mL syrup
e311.	AMOXICILLIN 250mg capsules
e3zu.	AMOXICILLIN 250mg/5mL s/f susp
e3zn.	AMOXICILLIN 250mg/5mL syrup
e312.	AMOXICILLIN 500mg capsules
e3zq.	AMOXICILLIN powder 3g/sachet
e31b.	AMOXIL 125mg/1.25mL paed susp
e315.	AMOXIL 250mg capsules
e316.	AMOXIL 500mg capsules
e3zo.	AMOXYCILLIN 125mg/1.25mL susp
e3zk.	AMOXYCILLIN 125mg/5mL s/f susp
e3zm.	AMOXYCILLIN 125mg/5mL syrup
e311.	AMOXYCILLIN 250mg capsules
e3zu.	AMOXYCILLIN 250mg/5mL s/f susp
e3zn.	AMOXYCILLIN 250mg/5mL syrup
e312.	AMOXYCILLIN 500mg capsules
e3zq.	AMOXYCILLIN powder 3g/sachet
e31k.	AUGMENTIN 125/31 in 5mL susp
e31P.	AUGMENTIN 250/62 in 5mL susp
e31h.	AUGMENTIN 375mg tablets
e31T.	AUGMENTIN 625mg tablets
e31Y.	AUGMENTIN-DUO 400/57in5mL susp
e61C.	CEFACLOR 125mg/5mL s/f susp
e615.	CEFACLOR 125mg/5mL suspension
e614.	CEFACLOR 250mg capsules
e61D.	CEFACLOR 250mg/5mL s/f susp
e616.	CEFACLOR 250mg/5mL suspension
e61a.	CEFACLOR 375mg m/r tablets
e618.	CEFACLOR 500mg capsules
e69..	CEFALEXIN
e695.	CEFALEXIN 125mg/5mL mixture

For peer review only

e69v.	CEFALEXIN 125mg/5mL syrup
e691.	CEFALEXIN 250mg capsules
e693.	CEFALEXIN 250mg tablets
e696.	CEFALEXIN 250mg/5mL mixture
e69w.	CEFALEXIN 250mg/5mL syrup
e692.	CEFALEXIN 500mg capsules
e694.	CEFALEXIN 500mg tablets
e697.	CEFALEXIN 500mg/5mL syrup
e69..	CEPHALEXIN
e695.	CEPHALEXIN 125mg/5mL mixture
e69v.	CEPHALEXIN 125mg/5mL syrup
e691.	CEPHALEXIN 250mg capsules
e693.	CEPHALEXIN 250mg tablets
e696.	CEPHALEXIN 250mg/5mL mixture
e69w.	CEPHALEXIN 250mg/5mL syrup
e692.	CEPHALEXIN 500mg capsules
e694.	CEPHALEXIN 500mg tablets
e697.	CEPHALEXIN 500mg/5mL syrup
e69f.	CEPOREX 125mg/5mL syrup
e698.	CEPOREX 250mg capsules
e69a.	CEPOREX 250mg tablets
e69g.	CEPOREX 250mg/5mL syrup
e699.	CEPOREX 500mg capsules
e69b.	CEPOREX 500mg tablets
e69h.	CEPOREX 500mg/5mL syrup
eg6..	CIPROFLOXACIN
eg67.	CIPROFLOXACIN 100mg tablets
eg6x.	CIPROFLOXACIN 250mg tablets
eg6w.	CIPROFLOXACIN 500mg tablets
eg69.	CIPROFLOXACIN 5g/100mL susp
eg6v.	CIPROFLOXACIN 750mg tablets
eg61.	CIPROXIN 250mg tablets
eg64.	CIPROXIN 500mg tablets
eg6A.	CIPROXIN 5g/100mL suspension
eg65.	CIPROXIN 750mg tablets
e31Q.	CO-AMOXICLAV 125/31mg/5mL susp
e31z.	CO-AMOXICLAV 250/62in5mL susp
e31t.	CO-AMOXICLAV 375mg tablets
e31X.	CO-AMOXICLAV 400/57mg susp
e31U.	CO-AMOXICLAV 625mg tablets
e612.	DISTACLOR 125mg/5mL suspension
e613.	DISTACLOR 250mg/5mL suspension
e617.	DISTACLOR 500mg capsules
e619.	DISTACLOR MR 375mg m/r tablets
ebI..	FOSFOMYCIN
eg14.	FURADANTIN 100mg tablets
eg13.	FURADANTIN 50mg tablets
eg1C.	GENFURA 100mg tablets

For peer review only

eg1B.	GENFURA 50mg tablets
e69m.	KEFLEX 125mg/5mL suspension
e69i.	KEFLEX 250mg capsules
e69k.	KEFLEX 250mg tablets
e69n.	KEFLEX 250mg/5mL suspension
e69j.	KEFLEX 500mg capsules
e69l.	KEFLEX 500mg tablets
eg1A.	MACROBID 100mg m/r capsules
eg17.	MACRODANTIN 100mg capsules
eg16.	MACRODANTIN 50mg capsules
eg1x.	NITROFURANT 25mg/5mL s/f susp
eg1..	NITROFURANTOIN
eg1z.	NITROFURANTOIN 100mg capsules
eg1w.	NITROFURANTOIN 100mg m/r caps
eg12.	NITROFURANTOIN 100mg tablets
eg1y.	NITROFURANTOIN 50mg capsules
eg11.	NITROFURANTOIN 50mg tablets
e52w.	PIVMECILLINAM HCL 200mg tabs
e521.	SELEXID 200mg tablets
ecc..	TRIMETHOPRIM
ecc1.	TRIMETHOPRIM 100mg tablets
ecc2.	TRIMETHOPRIM 200mg tablets
ecc4.	TRIMETHOPRIM 50mg/5mL s/f susp

For peer review only

Supplemental Material D - ICD-10 Codes for UTI to Determine Risk Periods

Code	Description
N10	Acute tubulo-interstitial nephritis
N12	Tubulo-interstitial nephritis, not specified as acute or chronic
N30.0	Acute cystitis
N30.8	Other cystitis
N30.9	Cystitis, unspecified
N39.0	Urinary tract infection, site not specified

For peer review only
